# Assessing progesterone receptor modulation in glioblastoma: from *in vitro* and animal model to a human pilot protocol

**DOI:** 10.1080/15384047.2025.2603095

**Published:** 2025-12-24

**Authors:** Denisse Arcos-Montoya, Patricia García-López, Talia Wegman-Ostrosky, Ignacio Camacho-Arroyo, Silvia Anahí Valdés-Rives, Claudia Bello-Alvarez, Joaquín Manjarrez-Marmolejo, Marisol De La Fuente-Granada, Alejandro Ordaz-Ramos, Daniela Ávila-González, Néstor Fabián Díaz, Carlos Fabricio Guadarrama-Rangel, Andrés Mauricio Bonilla Navarrete, Orwa Aboud, David F. Cantú-de-León, Bernardo Cacho-Díaz, Aliesha González-Arenas

**Affiliations:** a Departamento de Medicina Genómica y Toxicología Ambiental, Instituto de Investigaciones Biomédicas, Universidad Nacional Autónoma de México, Mexico City, Mexico; b Laboratorio de Fármaco-Oncología, Subdirección de Investigación Básica, Instituto Nacional de Cancerología, Mexico City, Mexico; c Unidad de Neuro-Oncología, Instituto Nacional de Cancerología, Mexico City, Mexico; d Unidad de Investigación en Reproducción Humana, Instituto Nacional de Perinatología-Facultad de Química, Universidad Nacional Autónoma de México, Mexico City, Mexico; e Laboratorio de Fisiología de la Formación Reticular, Unidad de Investigaciones Cerebrales, Instituto Nacional de Neurología y Neurocirugía, Mexico City, Mexico; f Departamento de Fisiología y Desarrollo Celular, Instituto Nacional de Perinatología, Ciudad de México, Mexico City, Mexico; g Department of Neurology and Neurosurgery, UC Davis Comprehensive Cancer Center, University of California Davis, Sacramento, CA, USA

**Keywords:** Glioblastoma, glioma, mifepristone, progesterone receptor, progesterone

## Abstract

**Background:**

Gliomas, including glioblastomas (GB) and high-grade astrocytomas (HGA), are the most common brain tumors in adults, with poor survival rates around 15 months. Hormonal factors, particularly progesterone receptor (PR) activation, promote tumor growth. Current treatment involves surgery, radiotherapy and chemotherapy (temozolomide), but survival rates remain low. Repurposing mifepristone (MF), a contraceptive drug, shows promise for GB treatment, warranting further study.

**Methods:**

PR expression in U87, U251 and C6 cell lines were assessed using immunofluorescence and Western Blot. PR isoforms were quantified by densitometry. Progesterone (P4) and 5α-dihydroprogesterone (5α-DHP) synthesis were evaluated using LC/MS. MF's effect on cell viability was determined by IC_50_ and IC_20_ values. Its impact on non-tumoral cells and 3D glioma sphere formation was also analyzed. The effects of *in situ* administration of MF were assessed *in vivo* using a rat model with C6 glioma implants. Clinical outcomes were evaluated in GB patients receiving MF alongside standard treatment.

**Results:**

PR was predominantly nuclear in all cell lines, with U87 showing the highest PR-B isoform levels. Only U251 synthesized 5α-DHP significantly. MF reduced viability in U251, U87 and C6 cells without affecting non-tumoral cells. Sphere formation efficiency decreased with MF treatment. In rats, MF reduced tumor volume dose-dependently. Clinically, MF improved patient survival from 165 to 588days and enhanced quality of life without severe adverse effects.

**Conclusion:**

MF effectively reduces GB cell viability, sphere formation efficacy and tumor volume. These findings support further investigation of MF as a therapeutic strategy in GB treatment.

**Précis (condensed abstract):**

Our research highlights the critical role PR in GB progression using *in vitro* and *in vivo* models. MF, a PR modulator, effectively reduced cell viability and sphere formation in cellular assays and significantly decreased tumor volume in an *in vivo* study. The pilot trial demonstrated the pharmacological safety of using MF as an adjuvant in GB treatment. Patients treated with MF showed a significant increase in survival, with an 80% survival rate at 1 year compared to 0% in those who were treated with the standard treatment.

## KEY POINTS

PR is highly expressed in GB cell lines.

MF reduces cell viability, sphere formation efficiency, and tumor volume in GB models and improves the quality of life in Mexican patients.

## IMPORTANCE OF THE STUDY

Understandin PR expression in GB is crucial due to its impact on tumor progression and response to therapies. This study elucidates differential PR isoforms expression and their functional implications in GB pathogenesis. MF, known for its PR-modulating properties, shows promising results in reducing GB cell viability and tumor volume *in vivo* without significant adverse effects. The study's findings suggest MF could serve as an effective adjuvant therapy for GB patients, potentially enhancing survival and improving quality of life. Furthermore, MF's ability to target PR without affecting non-tumoral cells underscores its specificity and therapeutic potential in GB management.

## Results

PR is highly expressed in GB cell lines. MF reduces cell viability and sphere formation efficiency.

*In vivo*, MF decreases tumor volume in a concentration-dependent manner.

MF improves the quality of life in Mexican patients.

This research provides critical insights into PR biology in GB and supports further clinical exploration of MF as a therapeutic adjunct in GB treatment strategies.

## 1 Background

Gliomas represent 81% of malignant central nervous system (CNS) tumors^1^, primarily affecting adults aged 28–84 with a peak incidence at 55–85 years old.^1^ In Mexico, the average age of diagnosis is 53.6 years old.^2^ These aggressive tumors encompass glioblastomas (GB) and high-grade astrocytomas (HGA), both of which are associated with 5-year survival rate of 6.9% and 27%, respectively.^3^ Male patients exhibit a predominance of 1.6:1 ratio^3^ and the annual incidence is reported as 3.6 cases per 100,000 individuals.^3^,^4^

Macroscopically, gliomas appear as poorly defined masses with grayish peripheries and yellowish necrotic centers. Molecularly, GBs are characterized by isocitrate dehydrogenase wild-type (*IDH*-wt) status, telomerase reverse transcriptase (*TERT*) promoter mutations, epidermal growth factor receptor (*EGFR*) amplification and chromosomal alterations,^4‐6^ while HGA are identified by mutations in isocitrate dehydrogenase 1 and 2 (*IDH1-2*), alpha thalassemia/mental retardation syndrome X-linked (*ATRX*), tumor protein p53 (*TP53*) and cyclin-dependent kinase inhibitors 2A and 2B (*CDK2A/B*).^7^

The development and progression of GB are driven by various factors, including dysregulation of growth factors and their receptors vascular endothelial growth factor (VEGF), platelet-derived growth factor (PDGF), epidermal growth factor receptor (EGFR),^8‐10^ altered signaling pathways (RTL/RAS/PI3K, RB, Wnt/β-catenin),^11‐13^ and changes in steroid hormone receptors, particularly the progesterone receptor (PR)^14‐18^ and the androgen receptor (AR).^19^ PR can be activated by progesterone (P4), which is synthesized in the brain mainly by astrocytes and neurons, diffusing through membranes to reach the tumor site.^20^ Additionally, GB-derived cell lines incubated with 3H-cholesterol have demonstrated the ability to synthesize P4.^21^ However, the potential for P4 production in GB cells under basal conditions remains largely unexplored.

*In vitro* and *in vivo* studies have shown that P4 at physiological concentrations promotes tumor growth by upregulating proteins related to proliferation and vascularization.^22^,^23^ Blocking PR activity with mifepristone (MF) decreases proliferation and invasion in glioma cell lines and reduces tumor volume in animal models using an intraperitoneal administration.^15^,^24‐26^ Notably, PR expression is higher in high-grade gliomas compared to lower-grade ones.^14^,^17^ Furthermore, P4 metabolites, such as dihydroprogesterone (5α-DHP) and allopregnanolone, have also been implicated in promoting GB progression.^27^

The primary treatment for malignant gliomas involves surgical tumor resection, followed by cycles of radiation therapy (RT) and chemotherapy with temozolomide (Tz), a protocol known as the Stupp regimen.^28^ Despite ongoing efforts to develop alternative treatments, the Stupp protocol remains the standard of care for these aggressive CNS tumors.^28^ Nevertheless, the 2-year survival rate is only 17.2%, with recurrences reported as early as 9 months posttreatment.^1^

Drug repurposing for new indications offers a promising strategy to accelerate the development of therapies for GB. MF is an approved contraceptive with well-characterized pharmacologic and toxicologic profiles. Studies in other cancers, including meningioma, ovarian and lung cancers, have demonstrated that MF can be safely administered at doses of 200 mg/day over extended periods.^29‐32^ Moreover, the concomitant use of MF with chemotherapy was previously evaluated in a phase I study for advanced breast cancer by Nanda et al. in 2016.^33^

This study aims to evaluate the effect of PR antagonism on GB progression using complementary approaches: *in vitro* cellular models (2D and 3D cultures), a murine *in vivo* model with intratumoral administration of MF, and an exploratory assessment of the safety of MF as an adjuvant treatment in patients with GB and HGA. We highlight the assessment of the optimal dose for *in situ* MF administration, which may support the development of MF-based wafers capable of reducing adverse effects, improving treatment specificity and enhancing dose control. Furthermore, we conducted a pilot study to evaluate the safety of combining MF with the Stupp protocol and to assess whether this combination improves the quality of life in a cohort of Mexican patients, expanding upon previously limited reports. Overall, this work represents a significant advance in GB therapy by presenting a target treatment strategy that integrates molecular insights, preclinical efficacy and clinical safety, with a focus on the Mexican population, while providing a scalable model for broader applications.

## 2 Results

### 2.1 Transcriptomic analysis reveals region-specific *PGR* expression in the human brain

Analysis of transcriptomic data from the Human Protein Atlas indicates that although *PGR* (PR coding gene) expression in the brain is modest compared to reproductive tissues, it does show region-specific distribution. The highest levels are observed in the hypothalamus (~19.4 nTPM), with moderate expression in the medulla oblongata, midbrain, pons and spinal cord (3.1–3.5 nTPM). Lower levels are found in the thalamus, basal ganglia and amygdala (2.2–2.8 nTPM), while there is minimal expression in the cortex, hippocampal formation, cerebellum and choroid plexus (1.1–1.6 nTPM) (Supplementary Table 1).

The regional specificity score (Tau = 0.56) within the brain indicates that *PGR* expression is most enriched in the hypothalamus but is still present in multiple subcortical and cortical regions, suggesting a diffuse yet potentially functional distribution. Expression in the brain is one to two orders of magnitude lower (0.2–10 nTPM) compared to classical hormone-responsive tissues (endometrium ~100 nTPM, cervix ~62 nTPM and ovary ~17 nTPM). Furthermore, the Atlas does not provide reliable immunohistochemistry data for the PR protein in the brain, possibly owing to technical limitations. Single-nuclei RNA-seq analysis further shows that *PGR* is classified as “group enriched” in vascular-associated cell types, including endothelial cells, fibroblasts, pericytes and vascular smooth muscle cells, while no neuronal or glial enrichment was reported (Supplementary Table 2). These findings suggest that PR expression in the brain may be primarily localized to non-neuronal compartments, particularly the neurovascular niche.

### 2.2 Distinct PR profiles in GB cell lines

**Figure 1.**
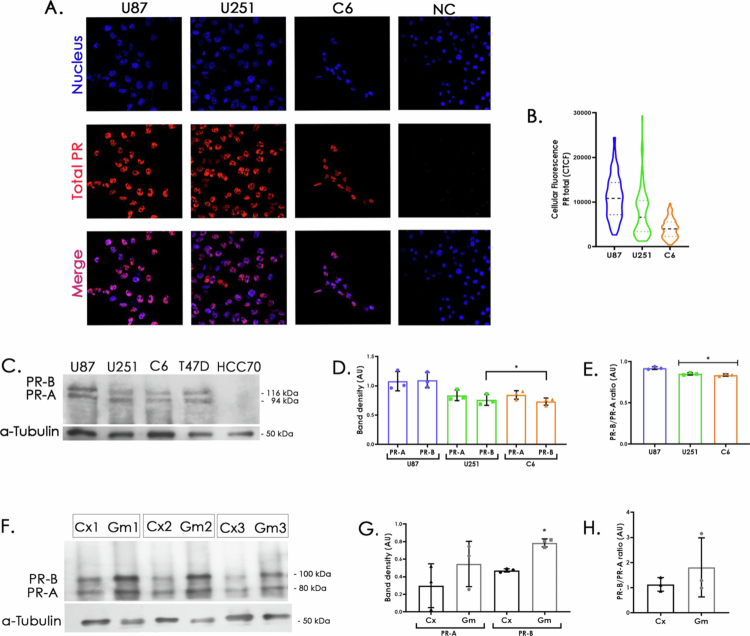
Characterization of PR content in GB cell cultures and tumors. A. Representative immunofluorescence for total PR (red), nuclei (Hoechst; blue), and merge in U87, U251 and C6 cell lines, and NC (negative control). B. The graph shows the quantification of the fluorescence intensity (CTCF) obtained for the PR (100 cells from 5 fields of 3 independent experiments), photographs were taken at 60× magnification (*p < 0.05, all were different). C. Representative Western Blot of PR isoforms (PR-A and PR-B) in human GB cell lines (U87 and U251) and murine glioma (C6), T47D and HCC70 breast cancer cell lines, were used as positive and negative controls, respectively. D. Densitometry analysis of western blot for PR isoforms and E. ratio. The graphs show the mean ± SD (*n* = 3) (*p < 0.05 vs U87). F. Western blot of murine glioma tissue (Gm) and non-tumor tissue (Cx) from the motor cortex contralateral to the tumor site from 3 independent rats. Both PR isoforms (PR-A and PR-B) are observed. G. Densitometric analysis for PR isoforms (PR-A and PR-B) *p < 0.05 vs. Cx. H. PR isoforms ratio.

The protein content of PR in U251 and U87 cell lines (derived from human GB), as well as in C6 (murine glioma), which shares key characteristics of GB,^34^ was characterized by immunofluorescence and Western Blot. Figure 1A shows that total PR is predominantly in the nucleus. The fluorescence intensity was quantified using corrected total cellular fluorescence (CTCF). As shown in Figure 1B, PR levels varied among the cell lines, with the highest expression observed in U87 and the lowest in C6. PR isoforms were further evaluated by Western Blot, distinguishing PR-B (114–116 kDa) and PR-A (92–97 kDa), using α-tubulin (50 kDa) as a loading control (Figure 1C). Densitometric analysis from three independent cultures (Figure 1D) revealed higher PR-B levels in U87 compared to U251 and C6, while PR-A levels were similar across all cell lines. Figure 1E shows the PR-B/PR-A ratio, confirming higher PR-B levels in U87 compared to U251 and C6 cell lines. Together, these data indicate that PR content varies among the cell lines, although subtle differences in PR-A expression cannot be excluded.

### 2.3 Elevated PR in glioma tissue and variable expression in human cancers

Once different PR isoform levels among cell lines were identified, we proceeded to examine PR expression *in vivo*. C6 cells were implanted into the motor cortex of three male Wistar rats. After four weeks, PR content was assessed in glioma tissue (Gm) and the contralateral, tumor-free hemisphere (Cx) by Western Blot (Figure 1F). Distinct expression patterns were observed. Densitometric analysis (Figure 1G) revealed significantly higher PR-B levels in tumor tissue compared to non-tumor tissue (Figure 1H), whereas PR-A levels were similar in both tissues.

To provide a broader context for PR expression in cancer, *PGR* was analyzed across multiple tumor types using mRNA data from patient samples available in The Cancer Genome Atlas (TCGA) database via the Xena Browser (https://xenabrowser.net/). This analysis revealed distinct expression patterns among glioma, endometrium, ovary, lung and breast tumors (Supplementary Figure 1), underscoring the variability of *PGR* expression across cancer types. Gliomas were found to exhibit the lowest *PGR* expression compared to other PR-dependent cancers. Nevertheless, in Mexican patients, PR protein levels have been reported to be higher in tumor tissue relative to non-tumor tissue.^14^

### 2.4 U251 cell line produces P4 and 5α-DHP

Considering that P4 and 5α-DHP act as natural ligands for PR and thus induce its transcriptional activity,^27^ it became essential to assess whether two of the cell lines utilized in this study possess the capacity to synthesize these steroids. We evaluated the cells' capability to synthesize them. To identify the P4 and 5α-DHP produced by the U87 and U251 cell lines, LC/MS was used. A dynamic multiple reaction monitoring (MRM) method was developed to detect specific precursors and product ions. The P4 and 5α-DHP were standardized (Supplementary Figures 2A and 3A, respectively). The retention times for P4 and 5α-DHP were 6.8 and 9.5 min, and the target fragment ions were m/z 315 → 97 for P4 and m/z 317 → 133 for 5α-DHP.

It can be observed that no peaks indicating the presence of P4 or 5α-DHP were found for the U87 cell line (Supplementary 2C and 3C). For U251 (Supplementary Figure 2B), a peak for P4 is shown; however, owing to its lower abundance compared to the standard, it could not be quantified. For 5α-DHP, we obtained a concentration of 2 µg/million cells (Supplementary Figure 3B). Based on this assay, it can be concluded that the U251 cell line synthesizes 5α-DHP in greater quantities than P4. Regarding the U87 cell line, no peaks indicating the presence of either P4 or 5α-DHP were found.

### 2.5 MF reduces the viability of GB tumor cells without affecting non-tumor cells

**Figure 2.**
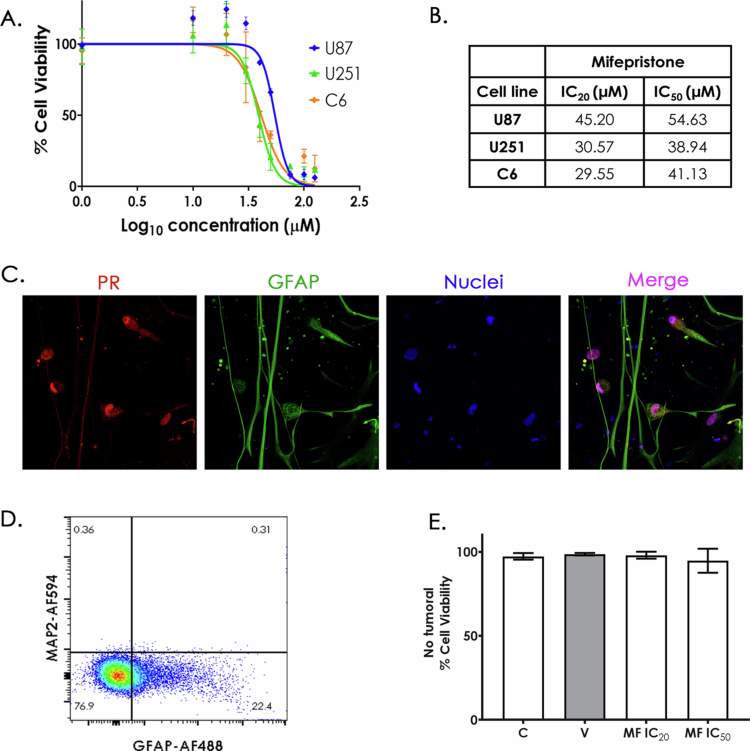
Mifepristone reduces cell viability in GB cell lines but not in a non-tumoral cell culture. A. Effects of increasing concentrations of MF in U251 (green), U87 (blue) and C6 (orange) cell viability. B. IC_20_ and IC_50_ values for each cell line. (*n* = 3, each dot represents mean ± SD), C. Immunofluorescence of differentiated astrocytes from hESCs showing PR (red) localized mainly in the nucleus, GFAP (green) marking astrocytes and Hoechst (blue) staining nuclei. D. Characterization of non-tumoral cell culture by flow cytometry, MAP2+ was used as a neuronal marker (0.36%), and GFAP+ for astrocytes identification (22.4%). E. Quantification of MF effect on non-tumoral culture, C: control, V: vehicle, MF: mifepristone (0.01% DMSO). Results are expressed as the mean ± SD, *n* = 3.

Concentration vs. viability curves were generated to determine the IC_50_ and IC_20_ of MF in U251, U87 and C6 cell lines (IC_50_/IC_20_: concentrations reducing viability by 20%/50%). Figure 2A shows the IC_20_ and IC_50_ for U251 (green), U87 (blue) and C6 (orange). The summarized results (Figure 2B) indicate values ranging between 30 µM and 50 µM. Variations in MF concentrations may be influenced by factors such as PR isoform expression and cell proliferation rates.

Afterwards, the effect of MF on non-tumoral cells was evaluated. A glial cell culture was derived from human embryonic stem cells (hESCs) according to Shie's protocol, with ethical approval 212250-212071 from the National Institute of Perinatology Ethics Committee. Glial fibrillary acidic protein (GFAP) and PR were detected in non-tumoral cells (Figure 2C). The merged image revealed that PR was predominantly localized in the nucleus. Notably, some GFAP-negative cells also expressed PR, indicating the presence of non-glial, non-tumoral populations that could theoretically respond to MF. To further identify these non-glial populations, flow cytometry was performed (Figure 2D), revealing 0.31% neuronal (MAP2+) and 22.4% glial (GFAP+) cells, with 76.9% likely representing glial progenitors.

Cells were treated with MF at IC_20_ and IC_50_ concentrations, and cell viability was assessed using a trypan blue exclusion assay. As shown in Figure 2E, no significant reduction in viability was observed after 72 h, indicating that MF does not adversely affect the non-tumoral cells present in this culture system.

### 2.6 MF decreases sphere formation efficiency

**Figure 3.**
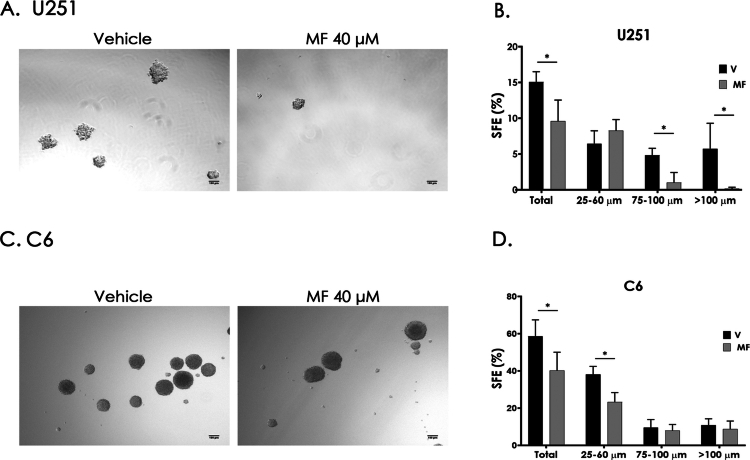
Mifepristone decreases sphere formation effiency in a 3D GB cell culture. Representative images of the effect of MF on the SFE of human glioblastoma cell line U251 and a murine glioma cell line (A and C). The total SFE in the spheres of 25–60 µm, 75–100 µm and ≥100 µm were plotted in U251 and C6 cells (B and D). Glioma spheres were treated with 40 µM MF (IC_50_) or vehicle (V, DMSO 0.01%) during seeding and then stimulated every 24 h for three consecutive days. On day ten, after sphere formation, their size distribution was determined. The results are expressed as the mean ± SD, *n* = 4; *p < 0.05.

To evaluate the effects of MF in a model that better recapitulates certain tumor characteristics, we generated 3D glioma sphere cultures. For the spheroid formation assay, we selected the U251 human GB cell line instead of U87 since U251 exhibited an IC_50_ for MF that was closest to that observed in the rat C6 glioma cell line. This similarity allowed a more consistent comparison between human and rat models. The sphere formation efficiency (SFE), defined as the ratio of formed spheres to seeded cells multiplied by 100, was assessed using the IC_50_ concentrations for U251 and C6 cell lines. Representative images are shown for U251 (Figure 3A) and C6 (Figure 3C) following MF treatment (40 µM). Quantification of SFE (Figure 3B and D) revealed a significant reduction in sphere numbers after MF exposure. In U251, spheres measuring 75–100 µm and >100 µm decreased, whereas in C6, spheres of 25–60 µm were diminished. These results suggest a decline in the population of cells capable of anchorage-independent growth, a characteristic associated with anoikis resistance and metastatic potential. Additionally, the observed decrease in sphere size may reflect alterations in cell proliferation or viability.

### 2.7 MF decreases GB tumor volume in a concentration-dependent manner in rats

**Figure 4.**
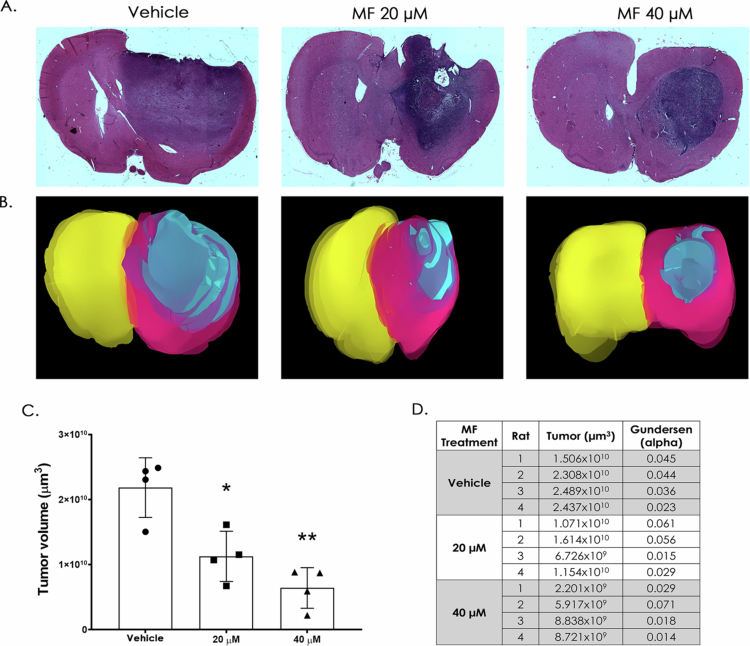
Mifepristone decreases GB tumor volume in an *in vivo* model. A. Representative images of brain sections stained with hematoxylin-eosin of each treated group; images are in a 10× magnification. B. 3D reconstruction of brain and tumoral volume, left and right hemispheres are shown in pink and yellow, respectively, and the tumor area is shown in blue. C. Quantification of the tumor volume (µm^3^). *n* = 4, presented as mean ± SD. (*p < 0.05 vs vehicle, **p < 0.01 vs vehicle). D. Tumoral volume assessed by stereological analysis, Gundersen *α* < 0.1 indicates an accurate representation of the sample in each brain.

Using the IC_50_ and IC_20_ values determined for the C6 cell line (Figure 2A and B), *in vivo* treatments were performed at 20 µM and 40 µM. Rats implanted with C6 cells received a 7-d MF treatment directly in the frontal cortex via cannula. Following treatment, animals were euthanized, and brain sections were stained with hematoxylin-eosin. Rats were monitored and weighed to ensure ethical compliance. Representative images show stained tumor lesions (purple) and unaffected areas (magenta) (Figure 4A). Tumor volume was quantified via 3D reconstruction (Figure 4B and Supplementary Videos), with hemispheres depicted in pink and yellow and the tumor areas in blue. Quantification (Figure 4C), demonstrated a significant reduction in tumor volume following MF treatment, showing a dose-dependent trend favoring 40 µM. Individual measurements (Figure 4D) exhibited Gundersen errors below 0.10, indicating accurate representation of the samples.

### 2.8 Adjuvant MF significantly prolongs survival in Mexican GB and HGA patients: a pilot study

Seven patients and seven controls were included in the pilot study: five patients with a histopathological diagnosis of GB and two patients with HGA (patient details are summarized in Table 1). All patients underwent the standard Stupp protocol (radiation therapy combined with temozolomide (Tz) chemotherapy). The seven patients concomitantly received MF. No ≥ grade 2 adverse events were reported, and the treatment adherence rate was 99%.

**Table 1.** Clinical-pathological data from patients in the pilot protocol (magenta-MF treatment) and historical control (blue).

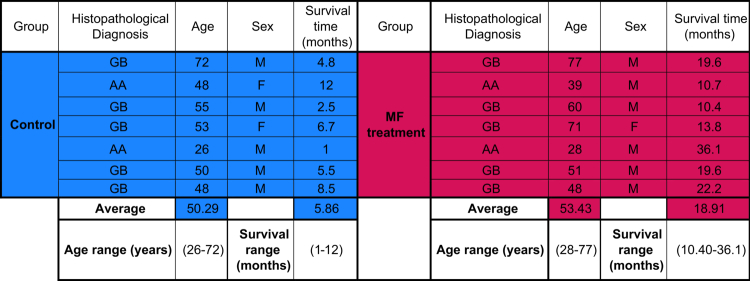

GB: glioblastoma, AA: anaplasic astrocytoma, M: male, F: female.

**Figure 5.**
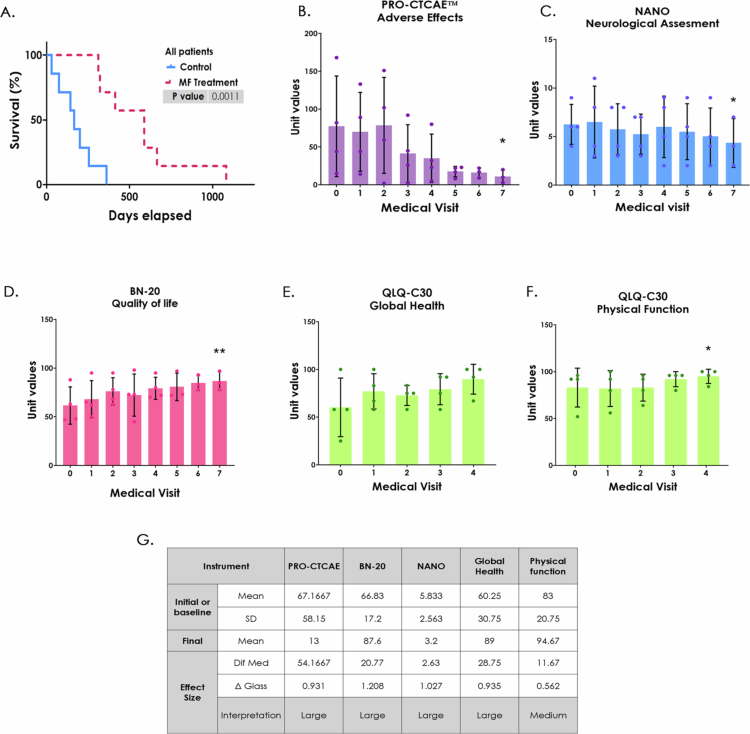
Survival analysis in patients treated with MF and the Stupp treatment and neuro-oncological evaluation questionnaires in patients treated with MF. A. Kaplan‒Meier survival curves of patients treated with the Stupp protocol (blue line—seven patients) and those with this protocol + MF (magenta line—seven patients). The difference was statistically significant (long-rank test, **p < 0.01) with a higher proportion of patients in the treated group surviving over time. B. PRO-CTCAE™ questionnaire for adverse effects. The higher scores represent a greater number and intensity of adverse effects. C. Neurological Assessment in Neuro-Oncology (NANO). The higher scores represent a greater neurological deficit. D. Patients' perception of the quality of life determined by EORTC QLQ-BN20 questionnaire. The higher scores represent a better perception of quality of life. Patients' perception measured by QLQ-C30 questionnaire, the higher scores represent a better perception of: E. global quality of life and F. physical function. Each patient is represented by a dot, (visit 0 = baseline value or start of treatment) Mean ± SD, *p < 0.05 and **p < 0.005 using Friedman test. G. determination of the effect size for clinical questionnaires using Delta Glass formula, Δ < 0.5 small effect, Δ = 0.5 medium effect and Δ > 0.5 large effect. Data from the historical control group did not include QoL information.

As shown in Table 1 and Supplementary Figure 4, there was no significant age difference between the control group (mean 50.29 years old, range 26–72) and the MF-treated group (mean 53.43 years old, range 28–77). Kaplan–Meier survival analysis revealed a significant increase in survival among patients treated with MF, with a median survival of 588 days compared to 165 days in the control group (Figure 5A). Cumulative survival analysis (Supplementary Table 3) at 1 and 2 years showed that MF treatment led to 71.4% survival in year 1 and 14.3% in year 2, whereas the control group exhibited 0% survival at both time end points.

When focusing on the five GB patients, survival improved from 165 to 588 days with MF treatment (Supplementary Figure 5A). Cumulative survival analysis (Supplementary Table 4) showed that 80% of GB patients in the MF group survived 1 year, compared to 0% in the control group. The mean of GB patients in the control group was 55.6 years old (range 48–72), while in the MF group, patients were 61.4 years old (range 48–77) in the MF group (Supplementary Figure 5B).

### 2.9 Adjuvant MF treatment enhances patients perceived quality of life

Various standardized questionnaires were used to assess patients' quality of life (QoL) throughout the treatment period, including PRO-CTCAE™, NANO, BN-20 and QLQ-C30 instruments. These assessments were conducted exclusively in the MF group. Data from the historical control group did not include QoL information since follow-up of this kind is not a routine practice.

For the PRO-CTCAE™ questionnaire (Figure 5B), a baseline score (Visit 0) was established prior to or at treatment initiation. Subsequent evaluations were conducted weekly during radiotherapy combined with Tz and MF, and monthly before each chemotherapy cycle. In this questionnaire, higher scores indicate a larger number and greater intensity of adverse effects reported by patients.

Overall, MF-treated patients showed scores with a clear downward trend and a significant reduction from the initial mean value (67.1) to the value at the end of treatment (13), showing a marked decrease in treatment-related adverse effects.

According to the NANO instrument, patients presented an average initial score of 5.8, which decreased to 3.2 by the end of MF treatment (Figure 5C), indicating an improvement in neurological performance and a reduction in functional limitations.

QoL was further assessed using the BN-20 instrument, where patients exhibited a progressive improvement in QoL perception after MF treatment (Figure 5D). The mean score increased from 66.83% at baseline to 87.6% post-treatment, representing a significant 20.77% overall improvement.

Similarly, the global health status, assessed through the QLQ-30 questionnaire, showed a 28.75% increase, from an initial mean of 60.25% to 89% after treatment (Figure 5E). Within this same instrument, physical functioning was also evaluated (Figure 5F), demonstrating a notable and significant enhancement, with scores rising from 83% at baseline to 94.67% after six months.

To assess the clinical relevance of these improvements, the Δ Glass formula, a standardized tool that measures the magnitude of treatment effects between two patient groups or, in this case, between pre- and post-treatment states, was applied. This approach allows for quantification of the clinical impact of MF treatment on patient outcomes, thus contributing to clinical decision-making.^35^,^36^

As summarized in Figure 5G, analysis using the Δ Glass formula revealed that four out of five evaluated parameters showed a large size effect, while physical function exhibited a medium effect size.

## 3 Discussion

This study analyzed PR expression in human GB cell lines and in tissue from an *in vivo* model, revealing isoform variations and P4 and 5α-DHP synthesis in U251 cells. PR-A and PR-B isoforms differ in their activation sites and gene regulation functions, with PR-B acting as a strong transcriptional activator.^37^ Although GB exhibits lower *PGR* (the gene encoding PR) mRNA expression than other tumor types (Supplementary Figure 1) and lower-grade brain tumors,^14^ it has been shown that in GB, PRs protein levels are increased in comparison to less malignant brain tumors and non-tumoral brain tissues.^14^

Analysis of publicly available transcriptomic data further supports our findings. According to the Human Protein Atlas, *PGR* expression in the human brain is modest compared to reproductive tissues but displays regional specificity. Single-nuclei RNA-seq data classify *PGR* as “group enriched” in vascular-associated cell types, while no enrichment is reported for neurons or glial populations. This pattern suggests that PR expression in the brain may be primarily localized to non-neuronal compartments, particularly within the neurovascular niche.

These observations align with our experimental data, which indicate that PR immunoreactivity in glioma tissues and cell lines, potentially reflecting receptor expression in tumor-associated endothelial or stromal cells rather than exclusively in transformed glial cells. Together, these findings reinforce the notion that the PR signaling axis in GB might influence not only tumor cell behavior but also the surrounding microenvironment through vascular or stromal interactions.

At physiological concentrations, P4-mediated PR activation promotes proliferation, vascularization and tumor growth.^22^,^23^ HPLC‒MS analyses confirmed endogenous P4 production in U251 but not in U87 cells; however, we cannot rule out the possibility that U87 cells possess the enzymatic machinery necessary for P4 synthesis or metabolism and may acquire P4 through alternative mechanisms. To improve sensitivity, we are considering to increase the number of cultured cells and to employ solid-phase microextraction (SPME) instead of liquid‒liquid extraction to enhance detection of low-abundance steroids. Previous studies indicate that astrocytes and other CNS cells may contribute to local P4 production, potentially activating PR in GB cells.^38^

Our analysis of PR isoforms ratio in U251 and C6 cell lines showed a predominance of the PR-A isoform over PR-B under *in vitro* conditions. Interestingly, this pattern appears to reverse *in vivo* when C6 cells are implanted, as shown in Figure 1. This shift underscores the dynamic regulation of PR receptor isoforms and highlights the importance of their thorough characterization. The differential expression may reflect the influence of the tumor environment on PR isoform regulation, which could have functional implications for tumor progression and therapeutic response. Notably, PR activity is not solely determined by ligand binding but can also be modulated through post-translational modifications, such as phosphorylation,^39^ which may further contribute to context-dependent differences in isoform functionality.

Blocking PR activity with MF resulted in a dose-dependent reduction in cell viability in both human and murine GB cell lines. Interestingly, despite differences in PR isoform expression, the IC_20_ and IC_50_ values were comparable across the different cell lines (30–55 µM). This suggests a consistent antiproliferative effect of MF, indicating a robust efficacy across cells with distinct molecular profiles, which is valuable for translational applications and may facilitate future testing in additional preclinical models. Previous GB studies testing MF used unique µM doses,^40^ therefore our IC_50_ assessment provides a more accurate measure of drug efficacy and enables more rational optimization of treatment strategies.^41^ In other studies, cancers such as endometrial cell lines (HEC-1-A and Ishikawa) have reported IC_50_ values of 37.3 µM (16 µg/ml) and 44.2 µM (19 µg/ml), respectively,^42^ and 20–40 µM in uveal melanoma cells.^43^ These values align with our results, reinforcing the consistency of our findings. In contrast, prior studies using breast cancer models^44‐46^ have shown that MF concentrations ranging from 10–100 nM can exert an antiproliferative effect, suggesting that the antiproliferative *in vitro* effect we observed here may not be exclusively PR-mediated.

Moreover, at our IC_50_ concentration, MF did not affect cell viability of non-tumoral glial cells derived from hESCs. This observation is particularly noteworthy, as it suggests that MF selectively targets tumor cells while sparing non-tumoral populations that also express PR, underscoring its potential specificity as an anti-glioma agent. However, given the widespread expression of PR across various tissues,^47^ and specifically in different CNS cell types,^20^ further research is required to fully understand its impact on normal cell function.

The 3D spheroid model, which better replicates tumor microenvironmental conditions such as oxygen and nutrient gradients, revealed a reduction in total SFE, which reflects the proportion of cells capable of anchorage-independent growth associated with anoikis resistance and metastatic potential. Sphere size reduction may also indicate decreased proliferation or viability.^48^,^49^ Nevertheless, *in vivo* models remain essential to evaluate complex processes that cannot be fully replicated *in vitro*, such as angiogenesis, invasion and immune response.^50^

MF reduces proliferation, migration and invasion in GB cell lines,^18^ MF also downregulates PR in various tissues and cancers, highlighting its therapeutic potential.^51^,^52^ *In vivo* studies further support MF's efficacy. In a rat model with glioma cells implanted in the hippocampus, systemic MF treatment in combination with Tz and radiotherapy reduced tumor volume and improved tumor boundaries, which are typically diffused in GB and hinder surgical resection. Additionally, systemic MF may enhance Tz by sensitizing tumor cells and inhibiting P-glycoprotein (P-gp), a key player in multidrug resistance.^25^,^53^

Mechanistically, in glioma xenografts, MF-induced cell death involves apoptosis via increased caspase-3 and Bax proteins and decreased Bcl-2 expression.^24^,^25^ MF has also been demonstrated to downregulate estrogen and progesterone receptors in endometrial cells, with stronger effects at higher doses.^51^ In the brain of postpartum estrus rats, MF reduced PR levels in the preoptic area, though not in the hypothalamus.^51^ Similar PR reductions were observed in breast and endometrial cancer,^52^,^54^ indicating its potential as a PR signaling modulator across cancers.

Unlike in previous models, here, the implant was performed in the motor cortex, which aligns with the most frequent site of GB occurrence and better recapitulates the invasive and migratory behavior of GB. Then, we implemented *in situ* MF administration in our model. This approach could reduce systemic adverse effects and enhance local drug efficacy by precise delivery to the tumor site.^55^ Using this method, we observed an ~50% reduction in tumor volume; however, whether *in situ* treatment in combination with Tz confers superior outcomes compared to systemic administration guarantees further studies.

Drug repurposing, exemplified by MF, a FDA-approved compound,^56^ provides a cost-effective and time-efficient alternative to traditional drug development, substantially reducing preclinical and early clinical phase costs.^57^,^58^ This strategy is particularly valuable in GB, where effective therapeutic advances are urgently needed.

Clinically, MF was tested as an adjuvant in Mexican GB patients receiving RT and Tz. Patients treated with MF exhibit a nearly 2-year survival rate, improved QoL, and no grade 2 or higher adverse effects (Figure 5). Grade 1 events, (mild nausea and transient lymphopenia), resolved upon completion of the treatment. QoL, assessed using EORTC, QLQ-BN20 and QLQ-C30 questionnaires, improved in patients receiving MF, collectively supporting the positive impact of MF treatment on patient-reported outcomes. Although MF was administered *in situ* in the *in vivo* model, this approach was not feasible in the pilot clinical study because of technical challenges (invasiveness and need for hospitalization). Nevertheless, localized administration may represent a promising future strategy, such as carmustine wafers.

Sex-related disparities have been consistently documented in several types of brain tumors, including gliomas. According to CBTRUS, the overall male-to-female incidence rate ratio (IRR) for gliomas is 1.47. A meta-analysis conducted in Latin American cohorts yielded an IRR of 1.39.^59^ One limitation of this study is common to all pilot trials, with only a small number of patients and a predominance of male participants, which may have introduced bias. Therefore, the results should be interpreted with caution. Nonetheless, this pilot study paves the way for future, larger clinical trials in which the sample size can be expanded, sex-based differences in adverse effects can be assessed, and clinical responses can be more robustly evaluated.

MF is a non-selective PR modulator; at high concentrations, it also antagonizes glucocorticoid receptor (GR) and AR, potentially offering additional therapeutic benefits.^60‐62^ Interestingly, despite its non-selectivity, our data suggest that MF's activity in GB may be primarily mediated through PR rather than GR. To further validate PR involvement in MF's mechanism, complementary pharmacological assays using the specific PR antagonist, ulipristal acetate (UPA), were performed (Supplementary Figure 6). UPA induced a dose-dependent decrease in cell viability (IC_50_ 77 and 53 µM for C6 and U251, respectively), supporting that a substantial proportion of MF's antiproliferative effects is PR-mediated. Nonetheless, GR signaling may also contribute to the overall effect of MF, so future studies to examine the relative contributions of PR, GR and AR pathways in glioma cells are guaranteed.

To further investigate whether the expression of either these two steroid receptors (PR and GR) have a key role in the survival of patients with GB, we conducted an *in-silico* analysis using TCGA patient data. We compared *PGR* and *NR3C1* (the gene encoding GR) expression and their association with survival. Survival analysis of GB patients revealed that high *PGR* expression was correlated with poorer overall survival, whereas *NR3C1* expression showed no prognostic value (Supplementary Figure 7A and 7B). According to CBTRUS report^3^ the mean survival for GB patients is 9 months, to further investigate if *PGR* expression could be related to long-term survival, a secondary analysis was performed. The effect was most pronounced during the 10–40-month follow-up (Supplementary Figure 7C and 7D). In contrast, no significant differences were observed for *NR3C1* expression. In one of our previous work,^14^ using similar analysis, we reported no differences in survival associated with *PGR* expression. This discrepancy is likely because of the fact that, at the time of the published data, the classification based on *IDH* status had not yet been established, and many samples—both *IDH*-mutant and *IDH*-wild-type samples—were grouped as GB. Consequently, some patients may have shown longer survival due to the beneficial effect of *IDH* mutations. In the current analysis, the sample selection was restricted to *IDH* wild-type cases.

Interestingly, dexamethasone (Dex), a widely used GR agonist in GB treatment for its anti-inflammatory effects, has been associated with increased proliferation, invasion, angiogenesis and reduced apoptosis, negatively affecting standard treatments such as Tz.^63^ A recent meta-analysis reported decreased survival in GB patients receiving high Dex doses, further highlighting the therapeutic relevance of steroid receptor antagonism in GB.^64^

Overall, this study provides compelling evidence for MF's repositioning as an adjuvant agent in GB therapy. Its tumor specificity, minimal adverse effects, and demonstrated improvements in survival and QoL underscore its potential to enhance current treatment protocols, particularly in the Mexican population. Further studies are needed to evaluate *in situ* MF administration in GB patients and to clarify the role of different steroid receptor antagonism (PR, AR and GR) in therapy.

## 4 Conclusions

Our research highlights PR's role in GB progression in cellular and animal models. MF effectively reduced cell viability and sphere formation efficiency in cellular assays and significantly decreased tumor volume in the *in vivo* study. The pilot protocol in patients demonstrated MF's safety and its potential to improve QoL as an adjuvant treatment for GB patients, improving their survival time. A larger randomized clinical trial is required to assess MF's efficacy, to test *in situ* treatment, and establish MF as a viable therapeutic option for GB.

## 5 Materials and methods

### 5.1 Gene expression analysis

To investigate the expression patterns of the PR, encoded by the *PGR* gene in both normal and tumor tissues, transcriptomic and clinical datasets were analyzed from public repositories.

#### 5.1.1 Transcriptomic data analysis from the Human Protein Atlas

Data on *PGR* expression across human brain regions were extracted from the Human Protein Atlas (HPA; www.proteinatlas.org) transcriptomic and single-nuclei RNA-seq datasets^65^,^66^. Normalized transcript levels (nTPM) and regional specificity scores (Tau) were obtained from the “Brain Atlas” section. Expression values were compared among brain regions and with representative hormone-responsive tissues (endometrium, cervix and ovary) to assess relative abundance. Cell-type enrichment was inferred from single-nuclei RNA-seq data to identify predominant cellular sources of *PGR* transcripts.

#### 5.1.2 Gene expression analysis in cancer datasets

*PGR* expression for patients data were obtained from Xena Browser using The Cancer Genome Atlas (TCGA) datasets to compare different cancer types. The data were processed to remove duplicates and normalized to the log_2_ scale. Statistical analysis included the Kruskal‒Wallis test, followed by Dunn's post-hoc test. Sample sizes were as follows: glioma (*n* = 153, including GB samples regardless of *IDH* status), endometrium (*n* = 180), ovary (*n* = 419), lung (*n* = 1011) and breast (*n* = 1092) tumors.

#### 5.1.3 Survival analysis in GB patients

For survival analysis, clinical and molecular data from GB patients were obtained from TCGA through the cBioPortal platform, using the cohort: TCGA, GDC. Patients were first classified according to the presence of *IDH* mutations, based on the mutational data provided by the database. Patients with no mutation on the *IDH* gene were classified as *IDH* wild type and retained for subsequent analysis.

RNA-seq Z-score normalized data were used to assess the expression levels of the progesterone receptor (*PGR*) and the glucocorticoid receptor (*NR3C1*). Patients were grouped into high- and low-expression groups according to quartiles of expression for each receptor. Using the clinical data provided by the platform, overall survival (OS) was evaluated across these groups. To reduce potential confounding effects of long-term survivors, analyses were restricted to a maximum follow-up of 40 months. According to the CBTRUS report^3^ the mean survival for GB patients is nine months to further investigate if *PGR* expression could be related with a long-term survival, a secondary analysis was performed considering survival outcomes between 10 and 40 months to explore potential differences masked by early survival. Kaplan‒Meier survival curves and log-rank tests were performed to compare OS between groups.

### 5.2 Cell cultures and treatments

All the cells were cultured in their respective growth media with the addition of 1% antibiotic-antimycotic (Penicillin-Streptomycin–10,000 U/µg/ml, *In vitro*, Mexico, Cat: A-01, Lot: 240307).

Human GB-derived cell lines U87 (HTB-14™ Lot: 70016790, ATCC®, USA, Appendix A), U251 (HTB-17™, Appendix B, tested by Instituto Nacional de Medicina Genómica by PCR, LDG-INMEGEN: 00451, LDG: 451-2), and murine glioma cell line C6 (CCL-107™ Lot: 58078536, ATCC®, USA, Appendix C) were cultured in DMEM medium with 10% FBS. T47D and HCC70 breast cancer cells were cultured in RPMI medium with 10% FBS.

Glial cells were obtained from human embryonic stem cells (hESC) using a modified protocol^67^ with Ethical Approval 212250-212071 for the National Institute of Perinatology Ethics Committee. Cells were maintained at 37 °C with 95% air and 5% CO_2_ (details in Supplementary methods). For characterization, cells were fixed and stained with monoclonal antibodies against neuronal (MAP2) and astrocytic (GFAP) markers. Then, they were analyzed by flow cytometry, using FlowJo software to generate histograms.

Cells were treated with MF/RU486 (M8046, Sigma) and UPA (see Supplementary methods) using 0.01% DMSO as a vehicle.

### 5.3 Western blotting

Proteins were electrophoresed on 12% SDS‒PAGE gels for the cell samples and 7.5% gels for tissue samples at 100 V, transferred to nitrocellulose membranes, and blocked with 5% BSA. Membranes were probed with anti-PR antibody (ab63605, Abcam), followed by an anti-rabbit secondary antibody conjugated to HRP. After stripping with acid glycine, membranes were reprobed with anti-α-tubulin antibody (sc-398103, Santa Cruz Biotechnologies) and incubated with Goat Anti-Mouse conjugated to HRP (ab6789, Abcam). Chemiluminescence signals were detected and quantified using ImageJ software (NIH) to correct differences in the protein loaded in each lane, and PR content was normalized to that of *α*-tubulin.

### 5.4 Immunofluorescence

Cells (8,000 per well) were plated on glass slides, fixed with 4% paraformaldehyde, and permeabilized with methanol. After blocking with 1% BSA, cells were incubated with anti-PR antibody (sc-166169, Santa Cruz Biotechnology) and anti-GFAP (ab68428, Abcam). After PBS rinses, cells were incubated with anti-mouse Alexa-Fluor-594-labeled and anti-rabbit Alexa-Fluor-488-labeled secondary antibodies (Invitrogen) and stained with Hoechst solution. Samples were visualized on an Olympus Bx43F microscope, and merged images were generated using ImageJ software. Negative controls (NC, without primary antibody) were performed for PR staining.

### 5.5 P4 and 5α-DHP production in GB cells

For the extraction of P4 and its 5α-reduced metabolites, 10 × 10^6^ U87 and U251 cells were used. The culture medium was removed, and cells were lysed by sonication (Ultrasonic Processor Model GEX130) in an ice bath with 10 kHz, 10 s pulses for 1.5 min (details in Supplementary methods). P4 and 5α-DHP were identified using Liquid Chromatography/Mass Spectrometry (LC/MS) with a dynamic multiple reaction monitoring (MRM) method. The conditions for the LC/MS study are in Supplementary Table 1. For the generation of standard curves, P4 (P8783, Sigma) and 5α-DPH (195886, MP Biomedicals, LLC) were used.

### 5.6 Viability assay

To establish the appropriate timing and cell density for the MTT assay, a growth curve was conducted (data not shown). U87, U251 and C6 cells (1500 per well) were seeded in a 96-well plate with DMEM and 10% FBS. After 24 h, MF or UPA in DMSO (0.01%) were added at various concentrations (1–125 µM). Based on the growth curve, 72 h was identified as the most suitable time point to evaluate the effects of MF on monolayer cells. After 72 h, MTT solution (5 mg/mL, thiazolyl Blue Tetrazolium Bromide, M5655, Sigma) was added, and plates were incubated at 37 °C and 5% CO_2_ for 3 h. Medium was removed, crystals dissolved with 200 µL DMSO, and absorbance at 570 nm and 630 nm was recorded. Cell viability percentage versus antagonist concentration was plotted, and IC_50_ and IC_20_ values were determined as drug concentrations reducing cell viability to 50% and 20% compared to controls.

For the nontumoral cell culture, trypan blue assay was performed, we used cell culture medium as the control and 0.01% DMSO as the vehicle.^68^

### 5.7 Sphere formation assay

U251 and C6 cells were cultured in ultra-low-attachment 96-well plates (Corning, NY, USA) at a density of 1 cell/µl (final volume 100 µl) to prevent cellular aggregation. The cells were maintained in serum-free neural stem cell medium (SFM) containing DMEM/F12 (Gibco, Thermo-Fisher Scientific, MA, USA) supplemented with B27 without vitamin A (20 µL/mL; Gibco, Thermo-Fisher Scientific, MA, USA), recombinant human epidermal growth factor (rhEGF; 20 ng/mL; Peprotech, NJ, USA), basic fibroblast growth factor (bFGF, 20 ng/mL; Preprotech, NJ, USA) and mix of antibiotics (amphotericin 0.00025 g/L, penicillin 0.0603 g/L and streptomycin 0.1 g/L; *In vitro* S.A., Mexico). Cells were treated with 40 µM MF (IC_50_) or vehicle during seeding and then treated every 24 h for three consecutive days. On day 10, the number of spheres formed and their size distributions were determined using photographs at 4× magnification and measurements with a Neubauer chamber. Sphere Formation Efficiency (SFE) was calculated based on sphere size distribution using the next formula:

SFE=(countedspheres)/(seededcells)x100

### 5.8 *In vivo* assay

All experiments were conducted in accordance with the rules for the care and use of experimental animals and approved by the Institutional Animal Care and Research Advisory Committee (CICUAL, ID 181, Appendix D) of the Universidad Nacional Autónoma de México, and complied with the “Technical specifications for the production, care and use of laboratory animals” published by the Secretaría de Agricultura in Mexico (SAGARPA, NOM-062-ZOO).

#### 5.8.1 Experimental animals

Male Wistar rats (170–190 g), free of specific pathogens and with no prior procedures, were obtained from the institutional Animal Models Unit of the Instituto de Investigaciones Biomédicas, UNAM, and were housed in polycarbonate cages under standard laboratory conditions (22 ± 2 °C; 12:12 h light-dark cycle) with *ad libitum* access to food and water. Upon arrival, rats underwent a 15-days acclimatization period before experimental procedures.

#### 5.8.2 Study design and experimental procedures

Animals were divided into four groups:

1. Untreated rats used solely for tumor tissue collection (*n* = 3).
2. Vehicle group receiving vehicle (1 µL propylene glycol; *n* = 4).
3. Treatment group receiving 20 µM MF (*n* = 4).
4. Treatment group receiving 40 µM MF (*n* = 4).

Thus, the total number of animals used in the study was 15. The experimental unit was a single animal.

Rats were intraperitoneally anesthetized with ketamine/xylazine (80 and 7 mg/kg, respectively) and positioned in a stereotactic frame. A craniotomy was performed at coordinates AP = 1.6 mm, L = 3.0 mm, V = 2.0 mm, relative with Bregma suture, and 1 × 10⁵ C6 murine glioma cells suspended in 2 µL of PBS were injected into the motor cortex via a 25-gauge stainless steel cannula connected to a Hamilton microsyringe. The skull opening was sealed with bone wax, local gentamicin was applied as an antibiotic, and the incision was closed. Baytril (enrofloxacin), a broad-spectrum antibacterial agent, was administered in the drinking water for 3 d post-surgery.

Ten days after implantation, the rats in the treatment groups received daily infusions of MF (20 or 40 µM) for seven consecutive days via a guide cannula placed at the tumor site. Vehicle animals received 1 µL of vehicle using the same procedure. At the end of treatment, animals were euthanized by pentobarbital sodium overdose (200 mg/kg, intraperitoneally; Pisabental®, PISA, Mexico), followed by transcardial perfusion with saline and 3.7% paraformaldehyde (PFA). Brains were collected, embedded in paraffin using a tissue processor (Histoquinet, Leica TP1020, Germany), and coronally sectioned at 6 µm using a microtome (Reichert-Jung 820-11, Germany). Sections were stained with hematoxylin and eosin (H&E) and analyzed using an Olympus BX51-WI microscope with a Disk Scanning Unit (DSU).

#### 5.8.3 Sample size

Sample size was initially determined using the resource equation method. After preliminary experimentation with four animals per group, the sample size was refined using the following formula for hypothesis-testing studies: *n* = 2DE^2^(Z^α/2^ + Z^β^)2/d^2^, where *SD* is the standard deviation, *d* is the effect size, Z^α/2^ is the standard normal deviation corresponding to the desired significance level, and Z^β^ is the standard normal deviation corresponding to the desired power. Based on these calculations, the final sample size per group was determined to be four.

#### 5.8.4 Inclusion and exclusion criteria

The inclusion criteria required animals to fall within the specified weight range (170–190 g) and to have no history of previous experimental procedures. Exclusion criteria included the presence of pain as determined by the Rat Grimace Scale and a reduction in body weight exceeding 20% of the initial value. No animals met the exclusion criteria, and thus, all animals were included in the final analysis.

#### 5.8.5 Randomization

Randomization was performed using Microsoft Excel by assigning each animal a random number using the = RAND() function. Animals were then sorted by ascending values and sequentially allocated into the treatment or control groups to ensure unbiased distribution.

#### 5.8.6 Control of confounding variables

To minimize potential confounders, animals from each group were distributed across cages and cage positions were rotated weekly on the rack. Treatments and outcome assessments were performed in the same order across all groups to reduce variability due to time of day.

#### 5.8.7 Blinding

Group allocation was concealed from the researcher responsible for daily animal monitoring and clinical assessment (e.g., weight loss and signs of pain). However, the person performing experimental surgeries and compound administration was aware of group assignments. Histological and data analyses were conducted by an independent, blind investigator.

#### 5.8.8 Outcome measures

Primary outcome measures included tumor volume (estimated stereologically using the Cavalieri principle) and protein expression by western blot. Additional outcomes included body weight monitoring and behavioral assessments (data not shown). The reliability of stereological volume estimates was ensured by calculating the Gundersen coefficient of error using Stereo Investigator software; values below 0.1 were considered acceptable for precision.

### 5.9 Pilot study in patients

A prospective open clinical trial was done at the Instituto Nacional de Cancerología (INCan) to assess the pharmacological safety and impact on survival and quality of life (QoL) of administering MF as an adjuvant treatment in GB patients. They signed an informed consent before the beginning of treatments. All interventions were reviewed and approved by the Institutional Scientific and Ethics Committees (018/033/CCI and CEI/1255/18) and by the Comisión Federal para la Prevención contra Riesgos Sanitarios (Cofepris OCF18001878-CAS/OR/8205/2018, Appendix E). Inclusion criteria were age >18 years, with no steroid use, and eligibility for Stupp regimen.^28^ As an exploratory pilot study, seven patients were included through consecutive, non-randomized enrollment. Exclusion criteria for women included pregnancy or being in a fertile-prone period, a history of uterine/vaginal hemorrhage, diagnosed endometrial cancer or meningioma, and a known allergy to MF. Exclusion criteria also included loss of contact with the patient or lack of information about the outcome and the use of Dex as adjuvant treatment.

The intervention consisted of orally administering MF at 200 mg/day 1 h before radiotherapy/temozolomide (RT/Tz) as described by Stupp et al.^28^ After maximal safe surgical removal of the tumor, patients received concurrent RT (60 Gy in 30 fractions) with Tz, an oral alkylating agent, administered at a dose of 75 mg/m² daily during radiation for six weeks. Following this, patients underwent adjuvant chemotherapy with Tz, typically at a dose of 150–200 mg/m² for 5 days every 28-days cycle, which continued for up to six cycles. The rationale for using a daily dose of 200 mg was based on its optimal biological effect and its widespread use in other studies.^69^ All patients had to start RT/Tz-MF within 6 weeks after surgery.

Follow-up included weekly visits and laboratory tests during RT/Tz-MF, followed by visits every 28 days until clinical progression or death. At each visit, QoL, a complete neurological exam, the Neurological Assessment in Neuro-Oncology scale (NANO), and adverse effects were assessed, measured according to CTCAE v5.0 criteria and PRO-CTCAE^TM^.

To assess drug safety during consultations, participants were asked to complete the PRO-CTCAE™ questionnaire, which consists of 81 questions assessing the presence and intensity of adverse effects. Responses ranged from: 0 none, 1 mild, 2 moderate, 3 intense, to 4 very intense.

The NANO (Neurologic Assessment in Neuro-Oncology),^70^ the BN-20 instrument (EORTC QLQ-BN20)^71^ and the EORTC Core Quality of Life Questionnaire instrument (QLQ-C30)^72^ were used to assess the QoL of patients. These assessments were conducted exclusively in the group of patients treated with MF, since data from the historical control group did not include QoL information, as routine follow-up of this kind is not commonly performed.

Data acquisition and analysis began at RT/Tz-MF initiation and continued monthly. Overall survival analysis used the initial neurosurgery as day 1. Empty MF blisters were collected and quantified at each visit to measure compliance. This study included only 2 year of follow-up.

For survival comparisons, historical control was used. Patients were matched 1:1 by histopathological diagnosis, age at diagnosis (±9 years old), gender, and treatment to ensure accurate comparisons and minimize bias. Inclusion criteria were histopathologically confirmed diagnosis, complete Stupp protocol treatment (resection, RT and QT with Tz), and complete patient information, including outcomes.

### 5.10 Statistical analysis

Statistical analysis was performed using GraphPad Prism 8.0.2. Prior to comparisons, data were assessed for normality using the Shapiro–Wilk test. Normally distributed data were analyzed using one-way ANOVA followed by Bonferroni (for IC_50_/IC_20_), Tukey (for protein expression), or Dunnett (for tumor volume) post hoc tests. Non-parametric data, such as neurological questionnaire scores, were analyzed using the Friedman test. Survival analysis was conducted using the Log-rank (Mantel–Cox) test. A *p*-value ≤ 0.05 was considered statistically significant. For each analysis, the exact number of patients, animals, or samples used is indicated in the figure legends.

## Supplementary Material

Supplemental material Supplementary Figure 1: Differential *PGR* gene expression across cancer types using TCGA data. *PGR* (PR coding gene) expression data were obtained from Xena Browser using TCGA datasets. The data were processed to remove duplicates and normalized to the log2 scale. Each dot represents an individual sample values. Statistical analysis was performed using the Kruskal‒Wallis test, followed by Dunn's post-hoc test. Horizontal bars indicate the mean ± SD. (*p < 0.0001 vs glioma). Sample sizes were as follows: glioma (*n* = 153), endometrium (*n* = 180), ovary (*n* = 419), lung (*n *= 1011) and breast (*n* = 1092).Supplementary Figure 2. Representative MRM chromatograms of P4 fragmentation. (m/z_3_) (315 → 97) with a retention time of 6.8 min A. Typical chromatograms of the P4 standard in blank samples. B. Corresponds to a chromatogram of P4 extracted from U251 cells. C. Corresponds to a chromatogram of P4 extracted from U87 cells.Supplementary Figure 3. Representative MRM chromatograms of 5α-dihydroprogesterone (5α-DHP) fragmentation (317 → 133), with a retention time of 9.4–9.5 min. A. Typical chromatograms of the 5α-DHP standard in blank samples. B. Chromatogram of 5α-DHP extracted from U251 cells. C. Chromatogram of 5α-DHP extracted from U87 cells. D, E. Calibration and normalized curves for analyte concentration determination. F. Concentration of 5α-DHP calculated using the linear equation.Supplementary Figure 4. Non-Significant differences in age between groups. *n* = 7 *p < 0.05.Supplementary Figure 5. Kaplan‒Meier curve for GB patients. A. patients treated with the Stupp protocol (blue line – 5 patients) and those treated with the Stupp protocol + MF (magenta line – 5 patients). The difference was statistically significant (long-rank test, **p < 0.01) with a higher proportion of patients in the treated group surviving over time. B non-significant differences in age between groups. *n* = 5 *p < 0.05.Supplementary Figure 6. Ulipristal acetate (UPA) reduces cell viability in GB cell lines in a dose-response manner. Effects of increasing concentrations of UPA in U251 (green) and C6 (orange) cells. Results are expressed as the mean ± SD, *n* = 4.Supplementary Figure 7. Overall survival analyses in patients with GB. A. Kaplan–Meier survival curves stratified by high versus low expression of the *PGR*. (*n* = 34 *PGR*-high and 34 *PGR*-low patients). A worse survival was observed in patients with high *PGR* expression. B. Kaplan‒Meier survival curves stratified by high versus low expression of *NR3C1* (*n* = 35 *NR3C1*-high and 34 *NR3C1*-low). No significant differences were detected. C. Kaplan‒Meier survival curves for *PGR* expression restricted to the 10-40 months follow-up interval (*n* = 17 *PGR*-high and 17 *PGR*-low patients). High *PGR* expression was significantly associated with poor survival. D. Kaplan‒Meier survival curves for *NR3C1* expression restricted to the 10-40 months follow-up interval (*n* = 17 *NR3C1*-high and 17 *NR3C1*-low patients). No significant differences were detected.Supplementary Table 1. LC/MS conditions to P4 identification. Available from: https://www.jove.com/es/v/10156/high-performance-liquid-chromatography-hplc.

Supplemental material Supplementary Tables

## Data Availability

The information on IC_20_ and IC_50_ concentrations for MF is available in the PubChem database. For the patient protocol, no new data was generated in support of this research.

## Abbreviations

AA: anaplastic astrocytoma
AE: adverse effect
AO: anaplastic oligodendroglioma
CNS: central nervous system
GB: glioblastoma
MF: mifepristone, also known as RU486
HGA: high‐grade astrocytomas
PR: progesterone receptor
P4: progesterone
RT: radiotherapy
Tz: temozolomide
