# Supplementary material for: Assessing progesterone receptor modulation in glioblastoma: from in vitro and animal model to a human pilot protocol
Source: Cancer Biol Ther. 2025 Dec 24;27(1):2603095. doi: 10.1080/15384047.2025.2603095 (PMC12758302; doi:10.1080/15384047.2025.2603095)
Supplement: Supplemental material — Supplementary Figure 1: Differential PGR gene expression across cancer types using TCGA data. PGR (PR coding gene) expression data were obtained from Xena Browser using TCGA datasets. The data were processed to remove duplicates and normalized to the log2 scale. Each dot represents an individual sample values. Statistical analysis was performed using the Kruskal‒Wallis test, followed by Dunn's post-hoc test. Horizontal bars indicate the mean ± SD. (*p < 0.0001 vs glioma). Sample sizes were as follows: glioma (n = 153), endometrium (n = 180), ovary (n = 419), lung (n = 1011) and breast (n = 1092).Supplementary Figure 2. Representative MRM chromatograms of P4 fragmentation. (m/z3) (315 → 97) with a retention time of 6.8 min A. Typical chromatograms of the P4 standard in blank samples. B. Corresponds to a chromatogram of P4 extracted from U251 cells. C. Corresponds to a chromatogram of P4 extracted from U87 cells.Supplementary Figure 3. Representative MRM chromatograms of 5α-dihydroprogesterone (5α-DHP) fragmentation (317 → 133), with a retention time of 9.4–9.5 min. A. Typical chromatograms of the 5α-DHP standard in blank samples. B. Chromatogram of 5α-DHP extracted from U251 cells. C. Chromatogram of 5α-DHP extracted from U87 cells. D, E. Calibration and normalized curves for analyte concentration determination. F. Concentration of 5α-DHP calculated using the linear equation.Supplementary Figure 4. Non-Significant differences in age between groups. n = 7 *p < 0.05.Supplementary Figure 5. Kaplan‒Meier curve for GB patients. A. patients treated with the Stupp protocol (blue line – 5 patients) and those treated with the Stupp protocol + MF (magenta line – 5 patients). The difference was statistically significant (long-rank test, **p < 0.01) with a higher proportion of patients in the treated group surviving over time. B non-significant differences in age between groups. n = 5 *p < 0.05.Supplementary Figure 6. Ulipristal acetate (UPA) reduces cell viability in GB cell li [file KCBT_A_2603095_SM8673.docx]

**Supplementary data**

**Supplementary Figure 1: Differential *PGR* gene expression across cancer types using TCGA Data.** *PGR* (PR coding gene) expression data were obtained from Xena Browser using TCGA datasets. The data were processed to remove duplicates and normalized to the log2 scale. Each dot represents individual sample values. Statistical analysis was performed using the Kruskal-Wallis test, followed by Dunn's post-hoc test. Horizontal bars indicate the mean ± SD. (*p < 0.0001 vs glioma). Sample sizes were as follows: glioma (n = 153), endometrium (n = 180), ovary (n = 419), lung (n = 1011), and breast (n = 1092).


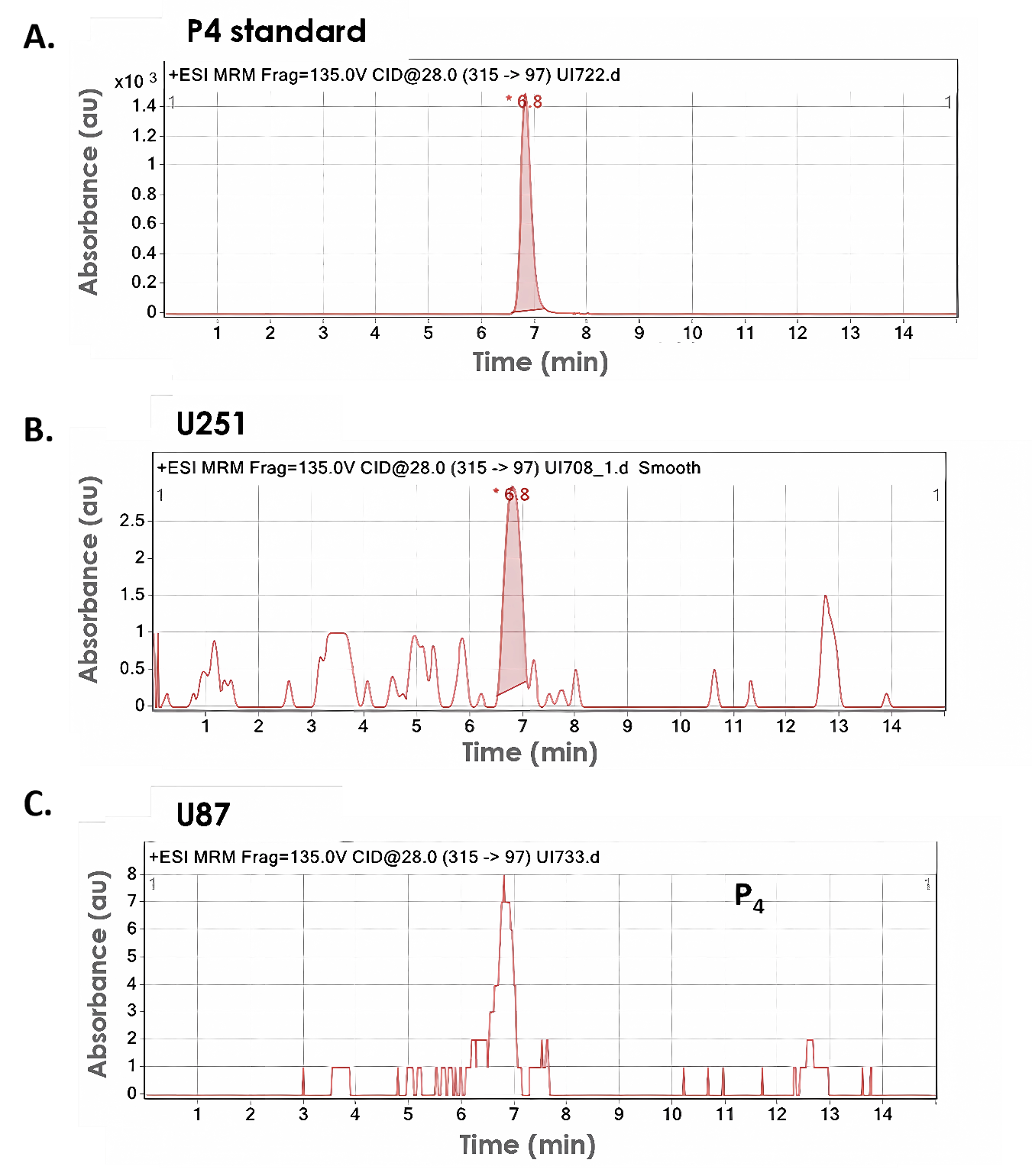


**Supplementary Figure 2. Representative MRM chromatograms of P4 fragmentation.** (m/z3) (315 → 97) with a retention time of 6.8 min **A.** Typical chromatograms of the P4 standard in blank samples. **B.** Corresponds to a chromatogram of P4 extracted from U251 cells. **C.** Corresponds to a chromatogram of P4 extracted from U87 cells.


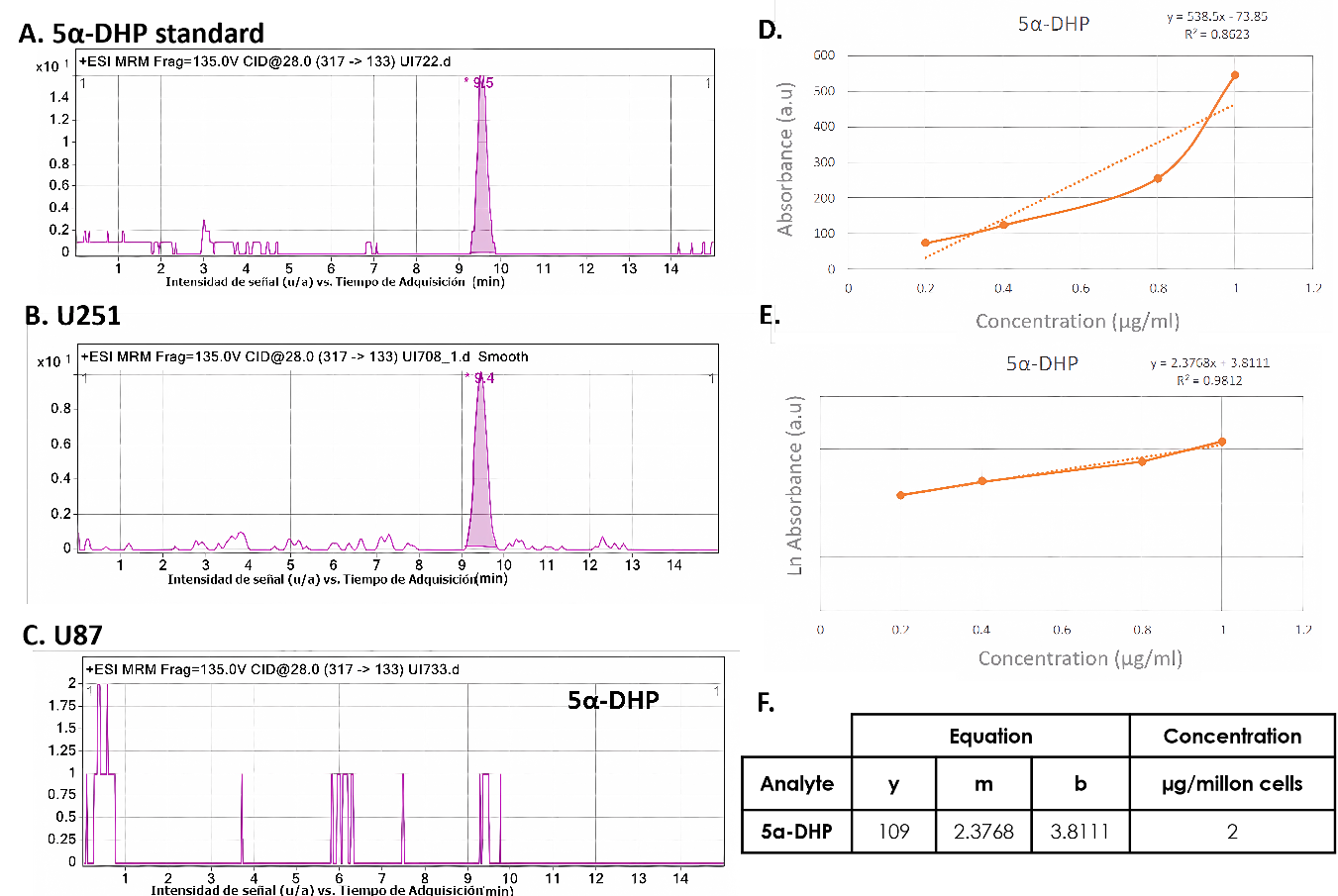


**Supplementary Figure 3. Representative MRM chromatograms of 5α-dihydroprogesterone (5α-DHP) fragmentation (317 → 133), with a retention time of 9.4–9.5 min.** **A.** Typical chromatograms of the 5α-DHP standard in blank samples. **B.** Chromatogram of 5α-DHP extracted from U251 cells. **C.** Chromatogram of 5α-DHP extracted from U87 cells. **D, E.** Calibration and normalized curves for analyte concentration determination. **F.** Concentration of 5α-DHP calculated using the linear equation.


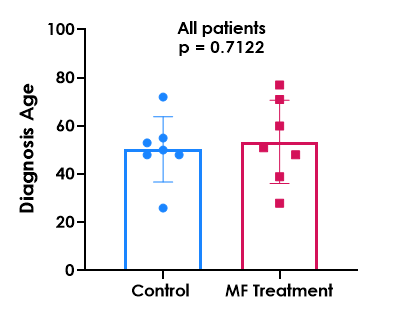


**Supplementary Figure 4. Non-Significant differences in age between groups.** n=7 *p<0.05.


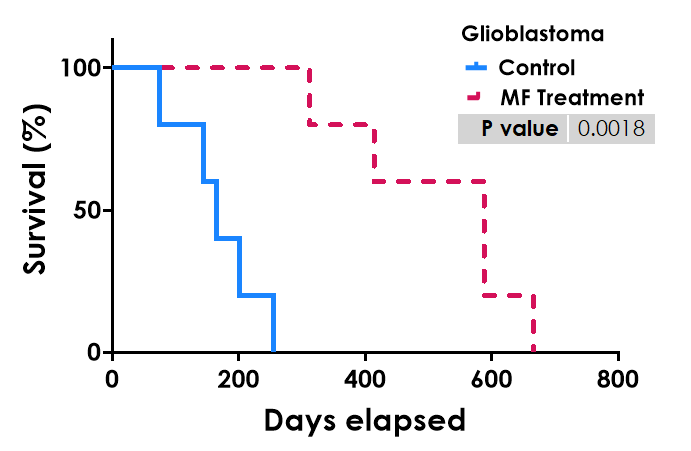

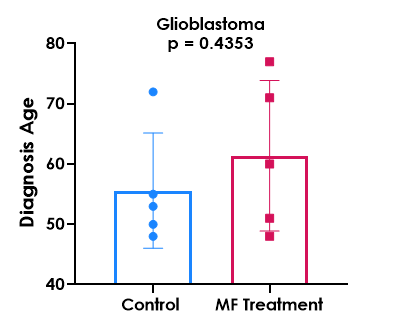


**P value = 0.0018**

**Supplementary Figure 5. Kaplan-Meier curve for GB patients.** **A.** Patients treated with the Stupp protocol (blue line – 5 patients) and those treated with the Stupp protocol + MF (magenta line – 5 patients). The difference was statistically significant (long-rank test, **p<0.01) with a higher proportion of patients in the treated group surviving over time. **B.** non-significant differences in age between groups. n=5 *p<0.05.


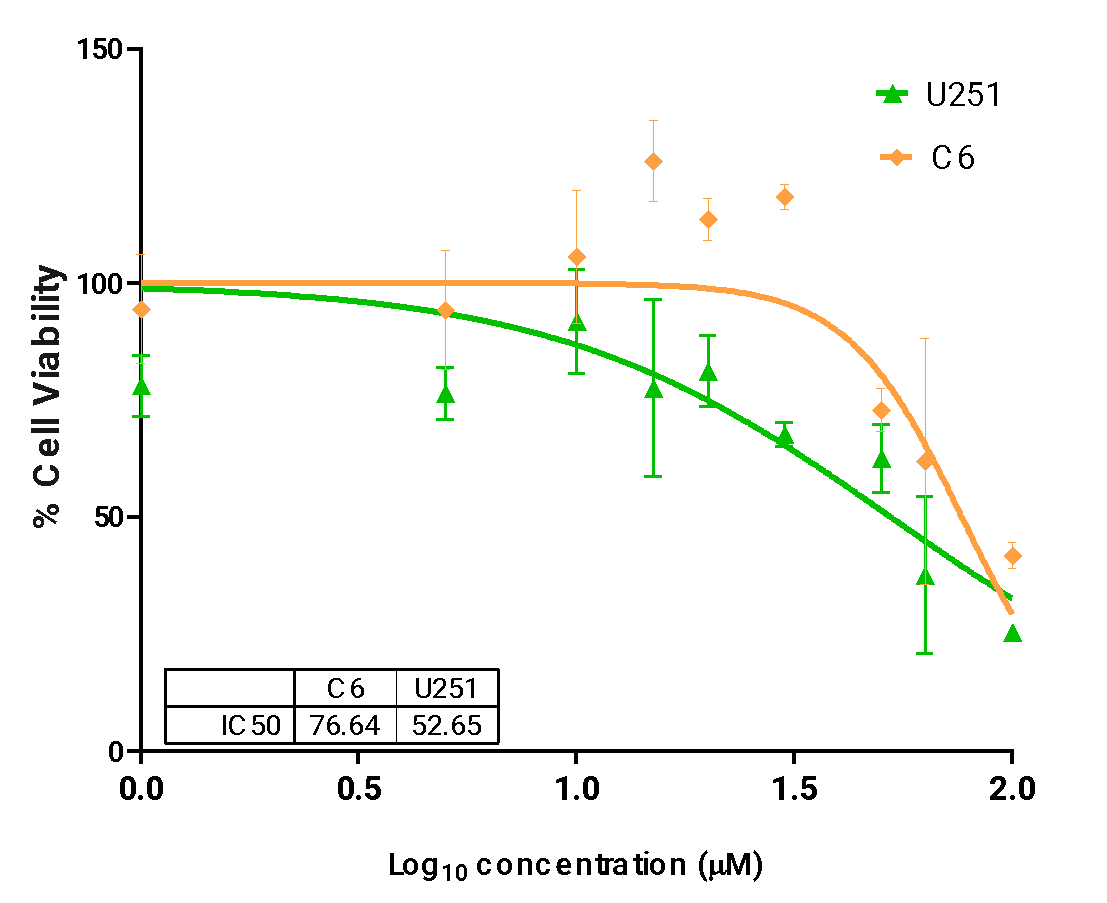


**Supplementary Figure 6. Ulipristal acetate reduces cell viability in GB cell lines in a dose-response manner.** Effects of increasing concentrations of ulipristal acetate in U251 (green) and C6 (orange) cells. Results are expressed as the mean ± SD, n = 4


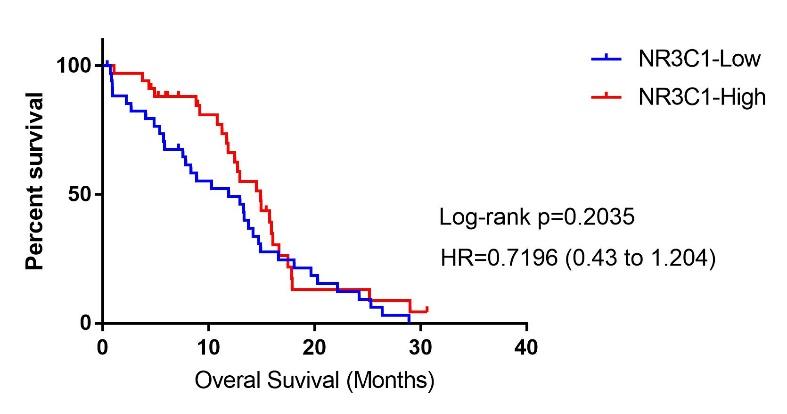

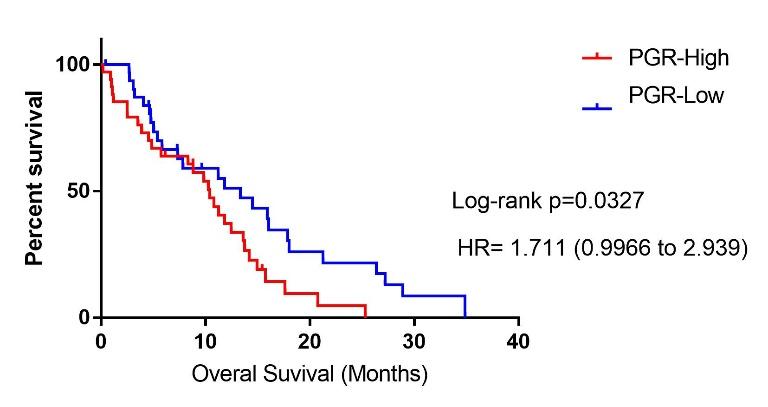


**A.**

**B.**


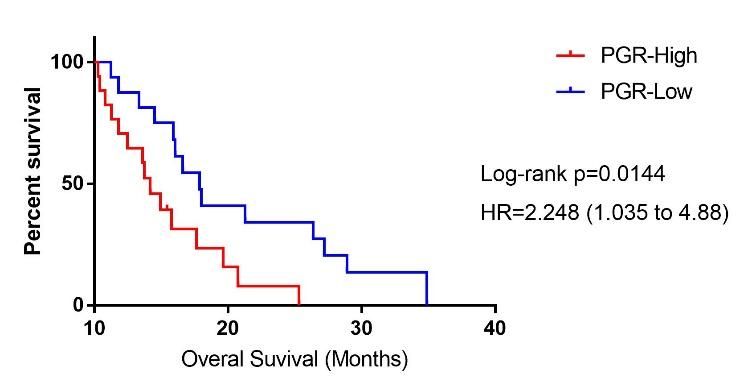


**C.**


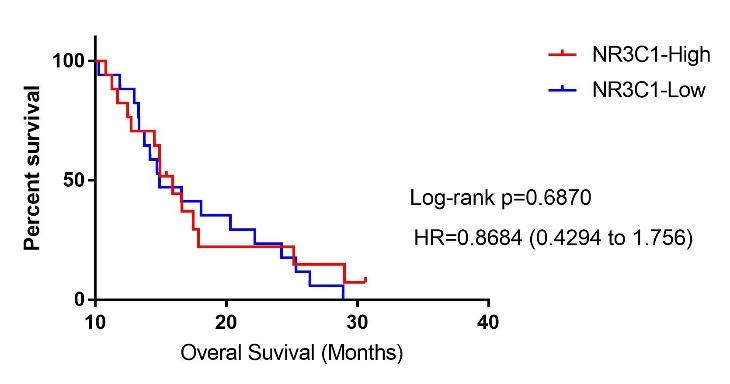


**D.**

**Supplementary Figure 7. Overall survival analyses in patients with GB.**  **A.** Kaplan-Meier survival curves stratified by high versus low expression of the *PGR*. (n=34 *PGR*-High and 34 *PGR*-Low patients). A worse survival was observed in patients with high *PGR* expression. **B.** Kaplan-Meier survival curves stratified by high versus low expression of the *NR3C1* (n=35 *NR3C1*-high and 34 *NR3C1*-low). No significant differences were detected. **C.** Kaplan-Meier survival curves for *PGR* expression restricted to the 10-40 months follow-up interval (n=17 *PGR*-high and 17 *PGR*-low patients). High *PGR* expression was significantly associated with worse survival. **D.** Kaplan-Meier survival curves for NR3C1 expression restricted to the 10-40 months follow-up interval (n=17 *NR3C1*-high and 17 *NR3C1*-low patients). No significant differences were detected.

**Supplementary methods**

- **hESC differentiation conditions**

The first day of differentiation was determined as day zero (D0). The FGF2 was removed from the medium, which was daily changed until D4, and two inhibitors of the SMAD pathway were supplemented (5 µM SB431642 and 1 µM Dorsomorphine). At D5, the cells were cultured with N2 medium (DMEM/F12, N2 supplement, GlutaMAX, 1% non-essential amino acids, 1% pyruvate of sodium, 500 µg/mL FBS, 1x penicillin and streptomycin, 0.1 mM 2-mercaptoetanol) supplemented with SB431542 and Dorsomorphine. The N2 medium was changed daily. At D12, cells were detached with dispase (0.875 mg/mL) and cultured on plates previously treated with poly-L-ornithine (10 µg/mL) and laminin (10 µg/mL) at a density of 50,000 cells/cm^2^ and with N2 medium supplemented with FGF2 (20 ng/mL) and Y27632 (a Rho-kinase inhibitor) (10 µM). At D14, the medium was changed with a mixture of N2 with B-27 medium (Neurobasal, B-27 supplement, GlutaMAX, 1X penicillin and streptomycin, 1% non-essential aminoacids) in a 1:1 ratio (N2B27) and supplemented with FGF2. For neural stem cell expansion, newly derived neural stem cells were passed with 50% Accutase every 4-5 days and cultured in N2B27 medium supplemented with FGF2 on poly-L-ornithine and laminin-treated plates. To initiate differentiation, cells were passed on Matrigel-treated plates and cultured with N2B27 without FGF2, and the medium was changed every four days. The cultures used for these experiments correspond to D100 differentiation cells.

- **Ulipristal acetate extraction**

Ulipristal was obtained from a pharmaceutical formulation (Femelle One, Delpharm Lillie S.A.S, Exeltis, France, batch ED144A). The tablet was pulverized using a mortar and solubilized in 1 mL of dimethyl sulfoxide (DMSO). The mixture was centrifuged at 15,000 g for 15 min to remove insoluble material, and the supernatant was recovered and filtered through a 0.22 µm syringe filter. A 63 mM stock solution was obtained from which serial dilutions were prepared for evaluation of cell viability using the MTT assay.

- **P4 and 5α-DHP extraction details**

The culture medium was removed, and cells were lysed by sonication (Ultrasonic Processor Model GEX130) in an ice bath with 10 kHz, 10s pulses for 1.5 minutes. Cell lysates (500 μL) were mixed with 500 μL of ethyl acetate, vigorously vortexed, and refrigerated overnight. After centrifugation at 14,000 rpm (4°C, 15 min), the organic phase was collected. The aqueous phase was re-extracted with 300 μL ethyl acetate, centrifuged, discarded, and the organic phase was evaporated at 30-45°C, and the residue was resuspended in 500 μL of ethanol.

**Supplementary Methods Table 1.** **LC/MS conditions to P4 identification.**

Available from: <https://www.jove.com/es/v/10156/high-performance-liquid-chromatography-hplc>

| **LC/MS analysis parameters** | |
| --- | --- |
| Ion source | Electrospray |
| Solvent A range (H_2_O) | 20% |
| Solvent B range (ACN) | 80% |
| Column | ZORBAX EXTEND-C185UM, 4.6x150MM |
| Gas temperature | 300 °C |
| Gas flow | 10 L/min |

**Appendix A**


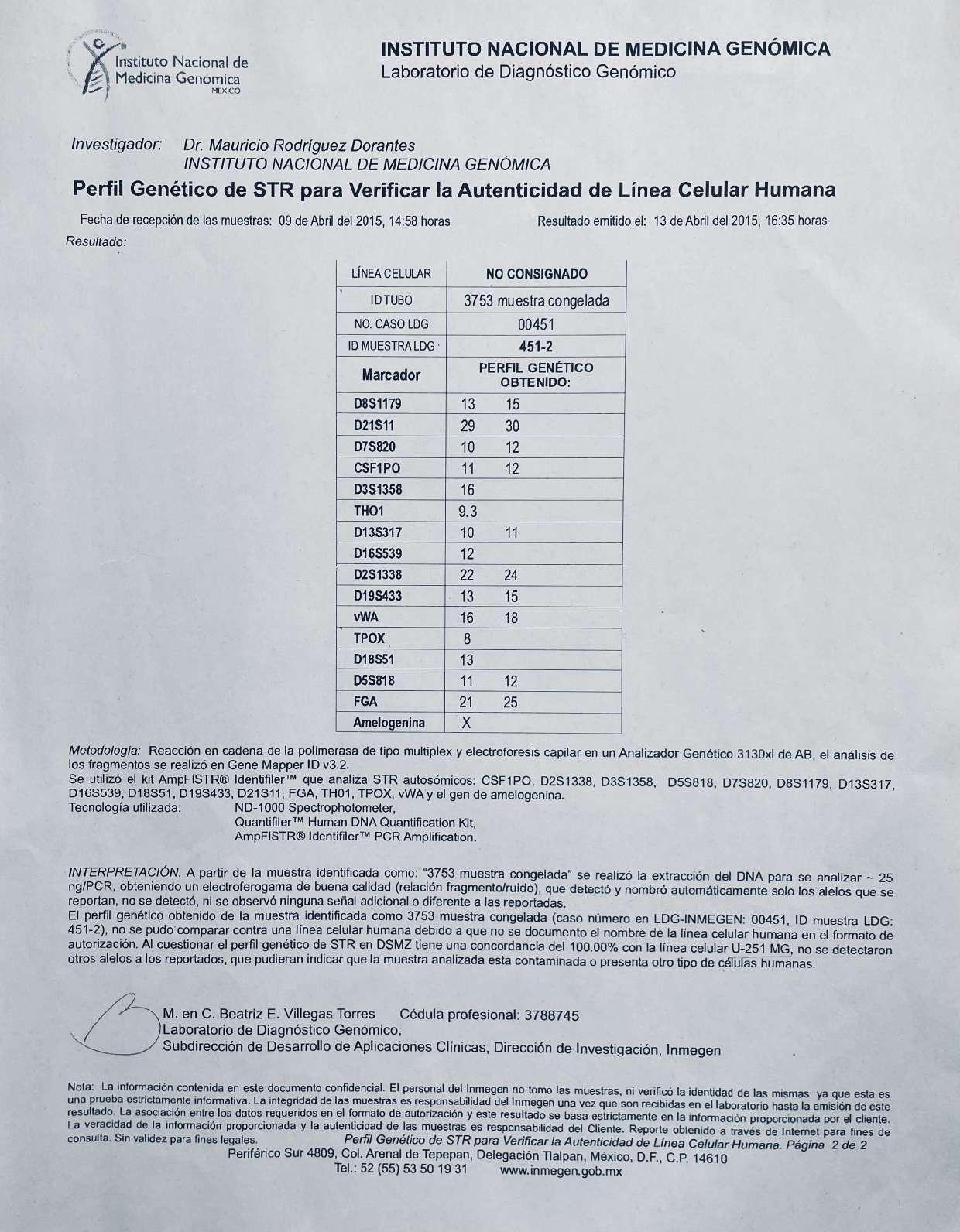


**Appendix B**


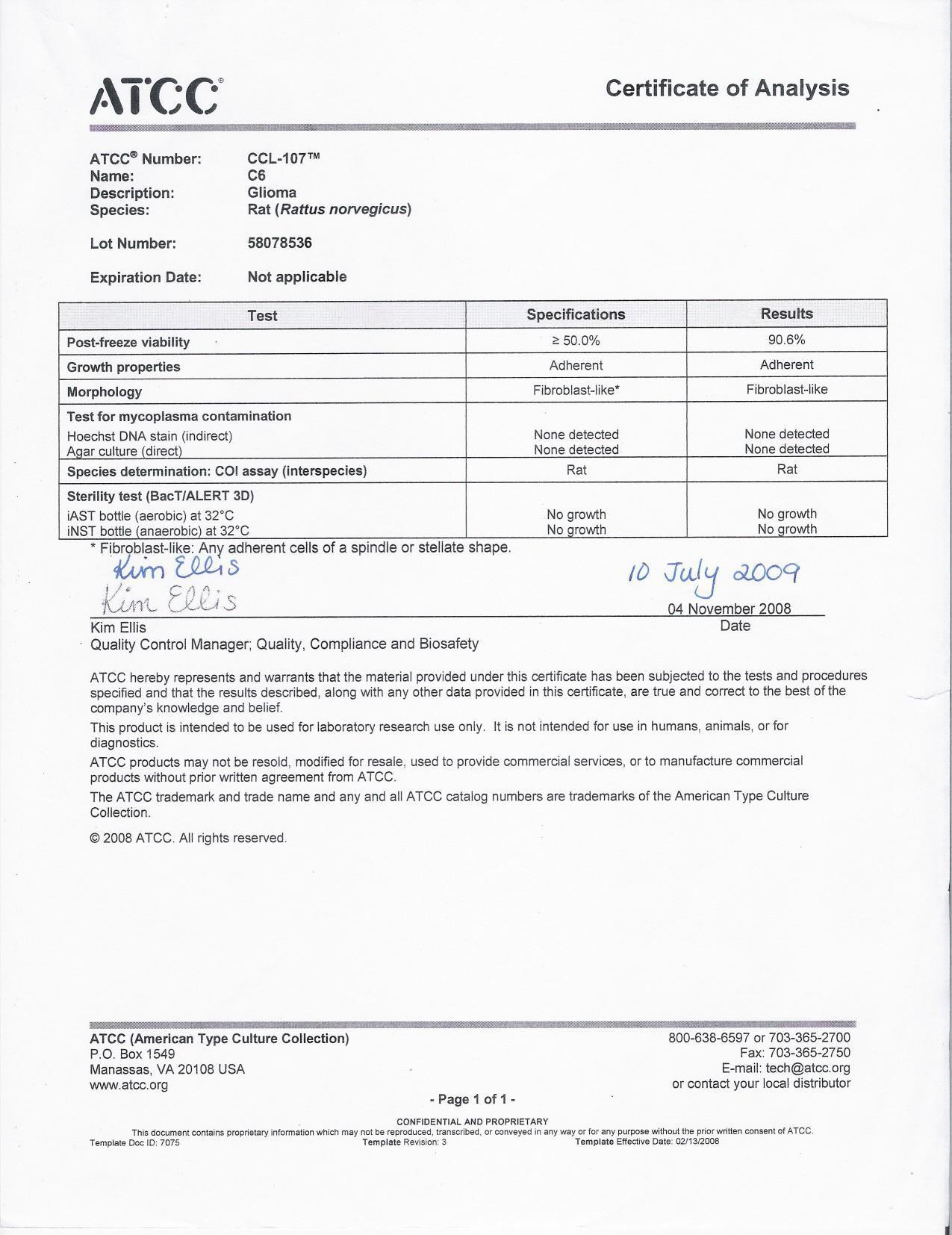


**Appendix C**


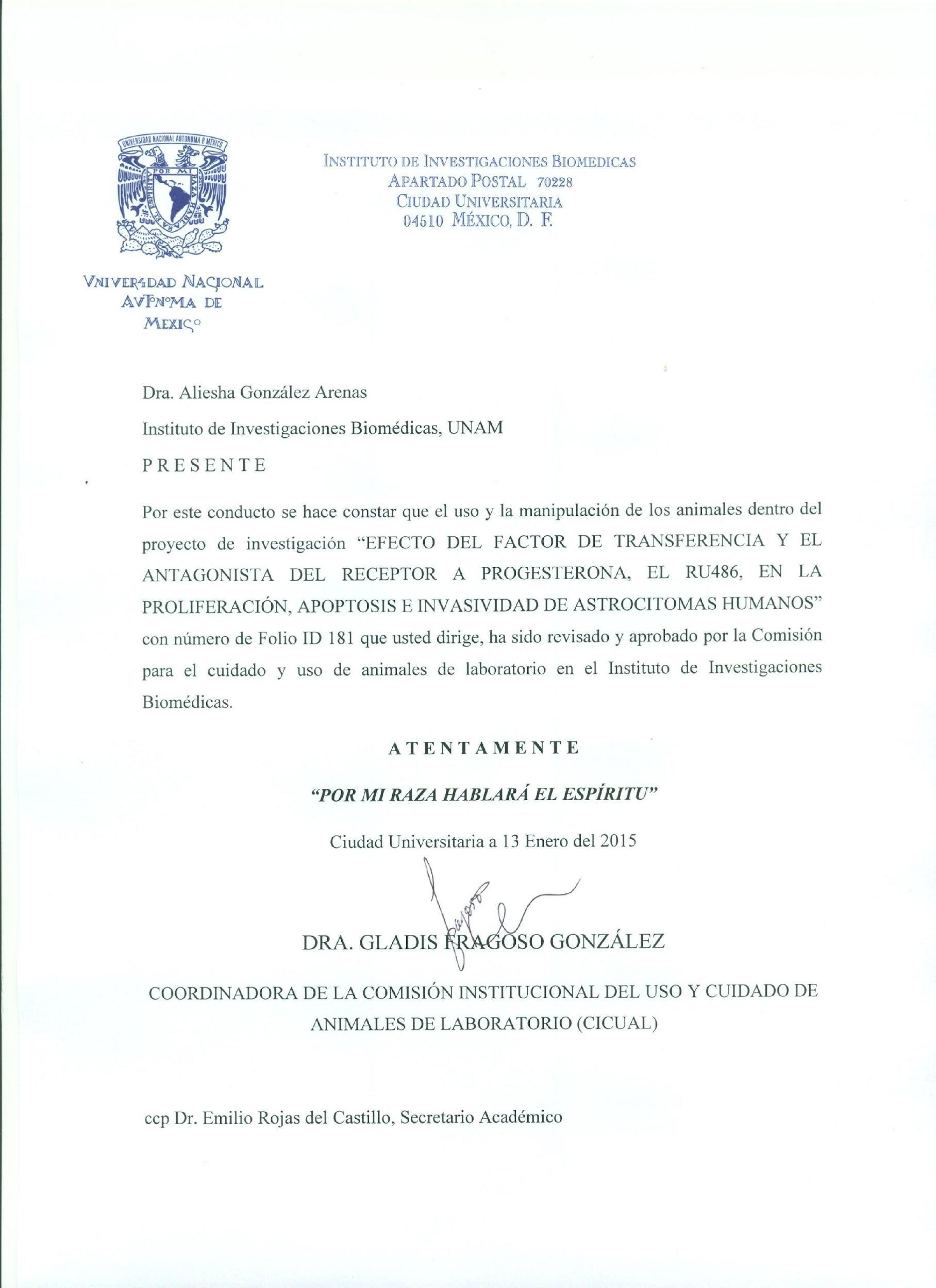


**Appendix D**


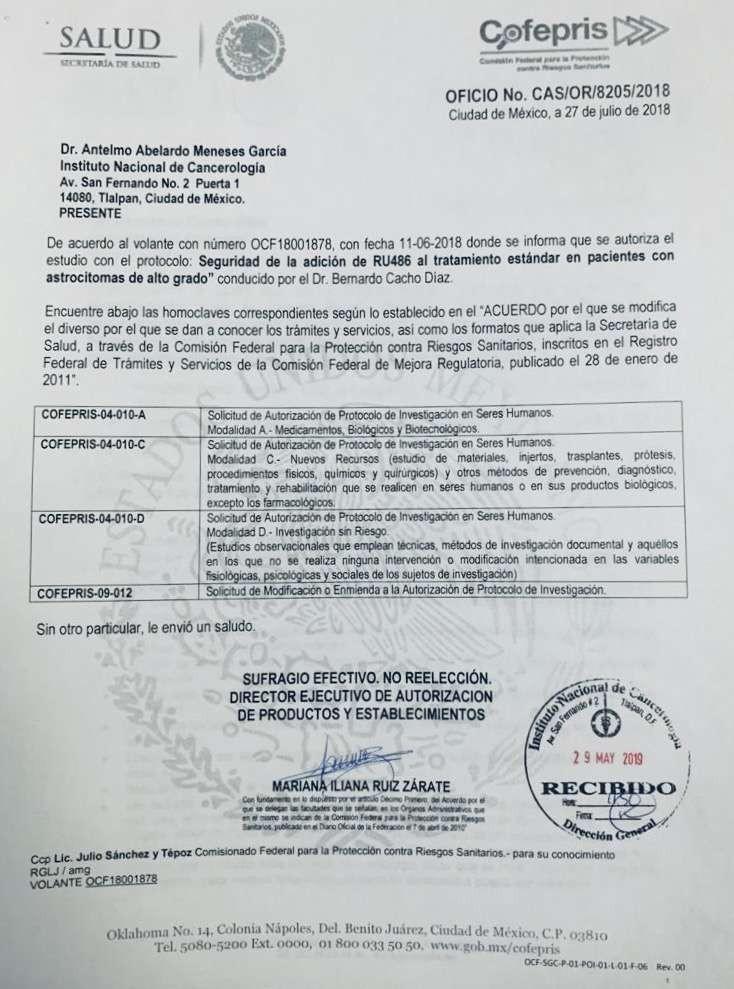


**Appendix E**
