# Supplementary material for: Assessing progesterone receptor modulation in glioblastoma: from in vitro and animal model to a human pilot protocol
Source: Cancer Biol Ther. 2025 Dec 24;27(1):2603095. doi: 10.1080/15384047.2025.2603095 (PMC12758302; doi:10.1080/15384047.2025.2603095)

**Supplementary Tables**

**Supplementary Table 1. *PGR* expression levels across human brain regions assessed by bulk RNA-seq (nTPM).**

| **Brain region**  **(bulk RNA-seq)** | **Expression (nTPM)** | **Relative abundance** |
| --- | --- | --- |
| Hypothalamus | 19.4 | Highest expression in brain |
| Medulla oblongata / Midbrain | 3.5 | Moderate |
| Pons | 3.4 | Moderate |
| Spinal cord | 3.1 | Moderate |
| Thalamus | 2.8 | Low |
| Basal ganglia | 2.4 | Low |
| Amygdala | 2.2 | Low |
| Cortex | 1.6 | Very low |
| Hippocampal formation | 1.3 | Very low |
| Cerebellum | 1.2 | Very low |
| Choroid plexus | 1.1 | Very low |

**Supplementary Table 2. *PGR* expression classification across human brain cell types assessed by single-nuclei RNA-seq.**

| **Cell type (single nuclei RNA-seq, brain)** | **Classification** | **Comment** |
| --- | --- | --- |
| Endothelial cells | Group enriched | Vascular compartment |
| Fibroblasts | Group enriched | Stromal compartment |
| Pericytes | Group enriched | Vascular support cells |
| Vascular smooth  muscle cells | Group enriched | Associated with brain vasculature |
| Neurons  (excitatory, inhibitory) | Not enriched | No significant detection reported |
| Glial cells (astrocytes, oligodendrocytes, microglia) | Not enriched | No significant detection reported |

**Supplementary Table 3. Cumulative survival analysis in GB and HGA patients.** Survival rate 1 year: Control 0% vs MF Treatment 71.4%, for 2 years: Control 0% and MF Treatment 14.3%. The gray lines highlight days nearly 1 and 2 years.


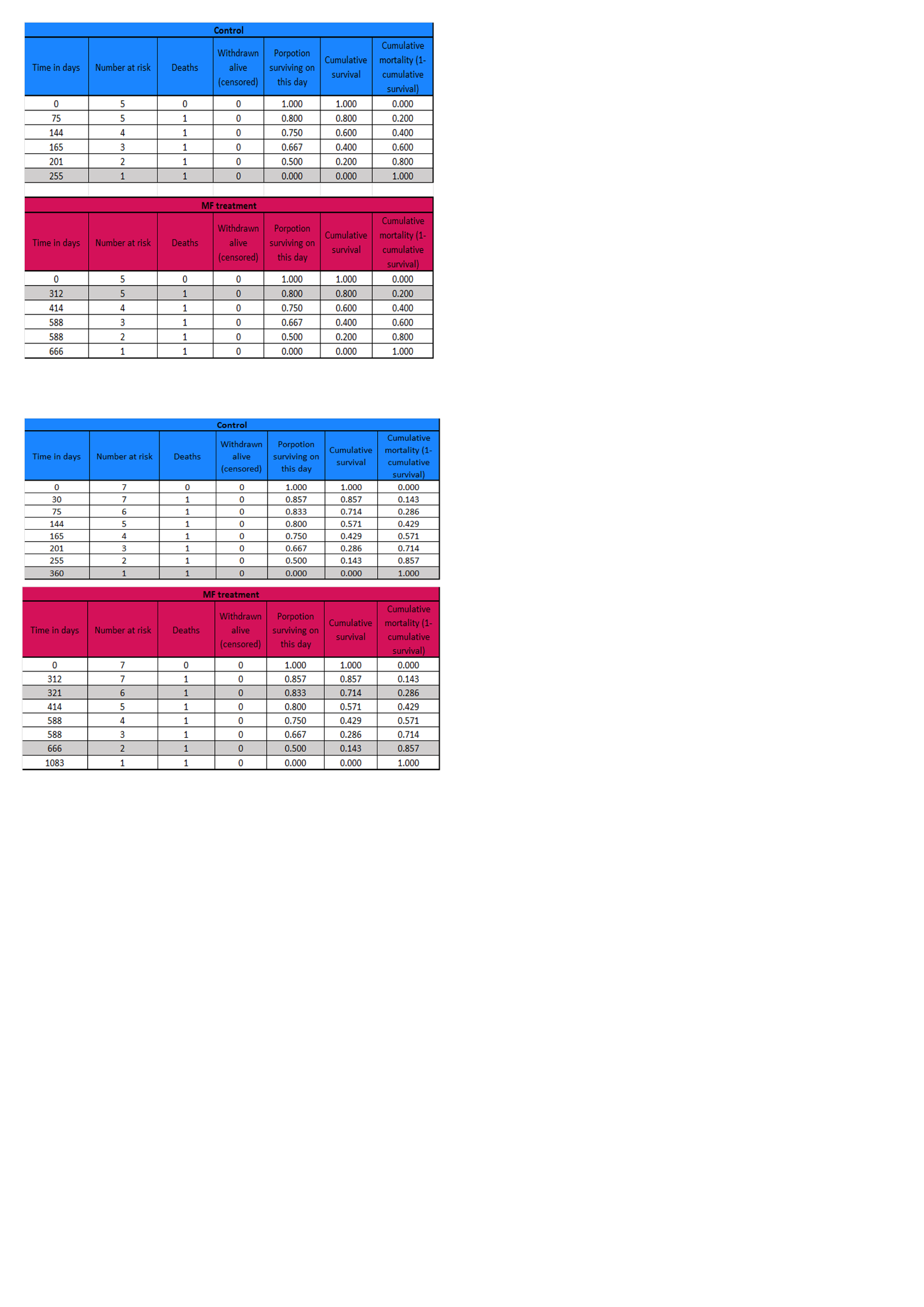


**Number at risk:** Number of subjects who are still under observation or at risk of experiencing the event of interest (death), **Withdrawn alive (censored):** Participants who are lost to follow-up before the event occurs. **Proportion of surviving on this day:** It is calculated as the number of surviving subjects divided by the total number at risk at that time point. **Cumulative survival:** It is calculated as the product of the proportions surviving at each previous time point. **Cumulative mortality:** It is calculated as 1 minus the cumulative survival probability at that time point.

**Supplementary Table 4. Cumulative survival analysis for GB patients.** Survival rate 1 year: Control 0% vs MF Treatment 80%. The days highlighted in gray represent nearly one year.


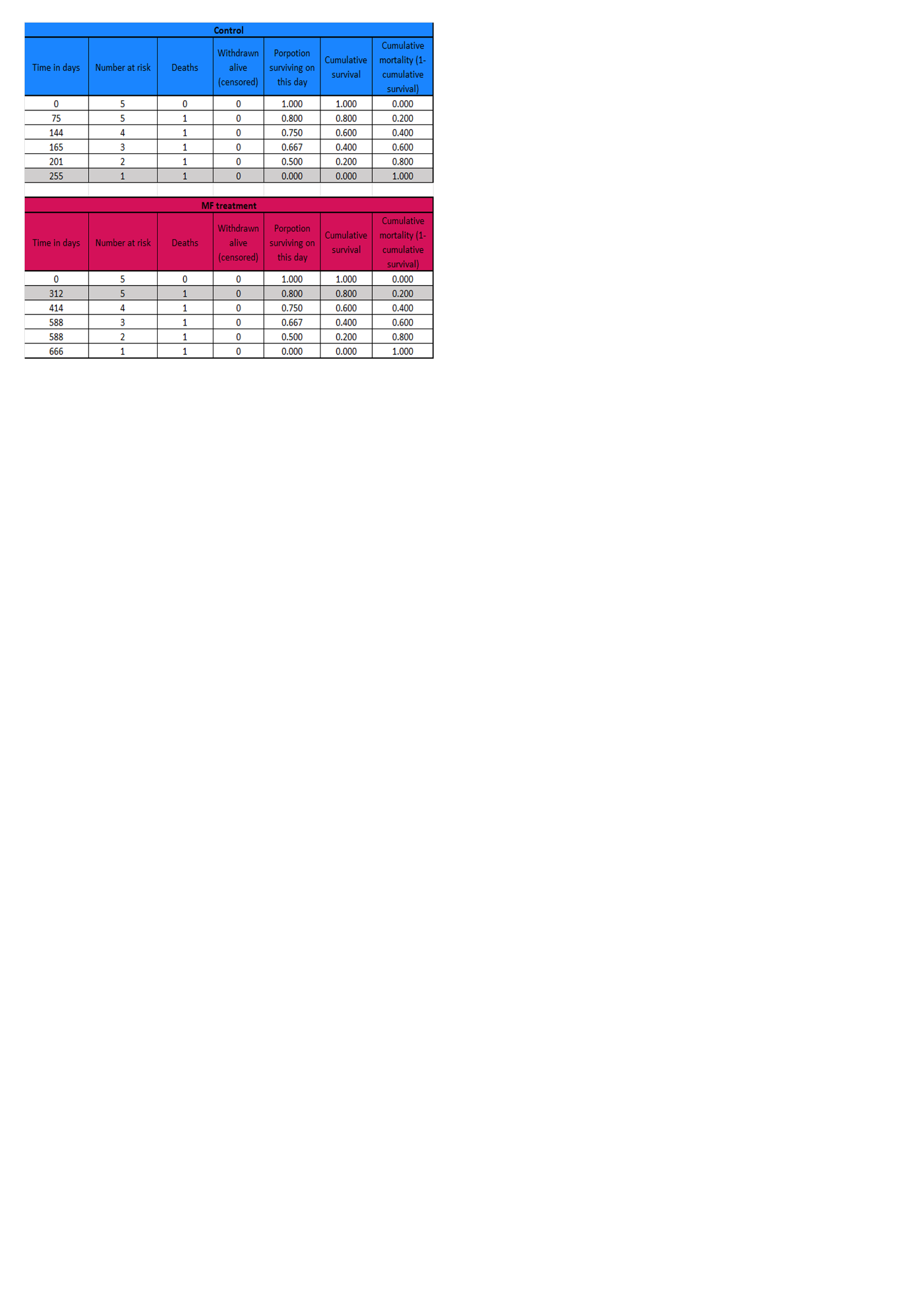

Supplement: Supplemental material — Supplementary Tables [file KCBT_A_2603095_SM8672.docx]
